# Multiple platform assessment of the EGF dependent transcriptome by microarray and deep tag sequencing analysis

**DOI:** 10.1186/1471-2164-12-326

**Published:** 2011-06-23

**Authors:** Franc Llorens, Manuela Hummel, Xavier Pastor, Anna Ferrer, Raquel Pluvinet, Ana Vivancos, Ester Castillo, Susana Iraola, Ana M Mosquera, Eva González, Juanjo Lozano, Matthew Ingham, Juliane C Dohm, Marc Noguera, Robert Kofler, Jose Antonio del Río, Mònica Bayés, Heinz Himmelbauer, Lauro Sumoy

**Affiliations:** 1 Bioinformatics and Genomics Program, Center for Genomic Regulation (CRG) - Universitat Pompeu Fabra (UPF), Barcelona, Spain; 2 Molecular and Cellular Neurobiotechnology Group, Institut de Bioenginyeria de Catalunya (IBEC)-Parc Científic de Barcelona, Barcelona, Spain; 3 Department of Cell Biology, University of Barcelona (UB), Barcelona, Spain; 4 Networked Biomedical Research Center for Neurodegenerative Diseases (CIBERNED), Madrid, Spain; 5 Microarray Unit, Genomics Core Facility, Center for Genomic Regulation (CRG) - Universitat Pompeu Fabra (UPF), Barcelona, Spain; 6 Genomics Core Facility, Center for Genomic Regulation (CRG) - Universitat Pompeu Fabra (UPF), Barcelona, Spain; 7 Genomics Unit, Institute of Predictive and Personalized Medicine of Cancer (IMPPC), Badalona, Spain; 8 Ultrasequencing Unit, Genomics Core Facility, Center for Genomic Regulation (CRG) - Universitat Pompeu Fabra (UPF), Barcelona, Spain; 9 Cancer Genomics Group, Vall d'Hebron Institute of Oncology, Barcelona, Spain; 10 Genes and Disease Program, Center for Genomic Regulation (CRG) - Universitat Pompeu Fabra (UPF), Barcelona, Spain; 11 Endocrinology Section, Hospital de Sant Joan de Deu, Esplugues de Llobregat, Spain; 12 Networked Biomedical Research Center for Diabetes and Associated Metabolic Diseases (CIBERDEM), Barcelona, Spain; 13 Servei de Inmunologia, Hospital Clínic i Provincial de Barcelona, Barcelona, Spain; 14 Institut d'Investigacions Biomèdiques August Pi i Sunyer, Barcelona, Spain; 15 Networked Biomedical Research Center for Hepatic and Digestive Diseases (CIBERHED), Barcelona, Spain; 16 Spanish National center for Genomic Analysis (CNAG), Barcelona, Spain; 17 Max Planck Institute for Molecular Genetics, Berlin, Germany; 18 IRSI-Caixa, Badalona, Spain; 19 Institute for Population Genetics, University of Veterinary Medicine, Vienna, Austria

## Abstract

**Background:**

Epidermal Growth Factor (EGF) is a key regulatory growth factor activating many processes relevant to normal development and disease, affecting cell proliferation and survival. Here we use a combined approach to study the EGF dependent transcriptome of HeLa cells by using multiple long oligonucleotide based microarray platforms (from Agilent, Operon, and Illumina) in combination with digital gene expression profiling (DGE) with the Illumina Genome Analyzer.

**Results:**

By applying a procedure for cross-platform data meta-analysis based on RankProd and GlobalAncova tests, we establish a well validated gene set with transcript levels altered after EGF treatment. We use this robust gene list to build higher order networks of gene interaction by interconnecting associated networks, supporting and extending the important role of the EGF signaling pathway in cancer. In addition, we find an entirely new set of genes previously unrelated to the currently accepted EGF associated cellular functions.

**Conclusions:**

We propose that the use of global genomic cross-validation derived from high content technologies (microarrays or deep sequencing) can be used to generate more reliable datasets. This approach should help to improve the confidence of downstream *in silico *functional inference analyses based on high content data.

## Background

Epidermal growth factor (EGF) is a key growth factor regulating cell survival. Through its binding to membrane receptors of the ERBB family, EGF activates an extensive signal transduction network that includes the PI3K/AKT, RAS/ERK and JAK/STAT pathways [1,2]. All these pathways predominantly lead to activation or inhibition of transcription factors affecting downstream mRNA transcription and regulating expression of both pro- and anti-apoptotic proteins, effectively blocking the apoptotic pathway. EGF-dependent signaling pathways are often dysfunctional in cancer, and targeted therapies that block EGF signaling have been successful in treating tumors [1,3,4].

Multiple approaches have been used to advance the knowledge of the cross-talk between signaling pathways, including the mapping of the complete EGF-dependent transcriptome and attempting to integrate it to build gene networks [5-13]. However, a comprehensive knowledge of the whole set of genes regulated by EGF stimulation is complicated by the fact that studies have been performed on different cell lines under a variety of treatment regimes (stimuli strength, length, timing). More importantly, in most cases results have not been validated by alternative methods on a whole genome scale, but only for a subset of genes. Two very thorough studies have used the HeLa cell line to establish the early response to EGF at the protein kinase phosphorylation level [14], and the transcriptional response profile in an extended time course treatment with EGF [4,11] aimed at investigating transcriptionally mediated feedback mechanisms that modulate response to EGF. This wealth of information makes HeLa cells an ideal experimental model to attempt to study the mechanisms of EGF signaling from a systems biology perspective.

Microarray studies have helped to uncover the transcriptional response to many intracellular signaling pathways that are perturbed by different drugs affecting growth factor responses, contributing to a better understanding of their mechanisms of action, and potentially leading to the identification of gene signatures correlated with drug efficacy and potential side effects [15-18]. Validation of microarray results by alternative methods is usually performed for genes of interest in order to distinguish true positives from the false positives expected from the inherent noise in highly multiplexed hybridization based technologies. The need for validation comes from the unavoidable fact that in microarray based hybridization assays there is always some degree of cross-hybridization to be accounted for, which may vary depending on the hybridization conditions as well as specific probe properties, such as sequence, length and GC content. The use of multiple microarray platforms in a single study could in principle be exploited as an alternative method to RT-PCR for global validation of changes in gene expression [19], and to confirm the detection changes in gene expression, although microarrays suffer from compression artifacts resulting in a lack of linearity relative to RT-PCR in the magnitudes of fold change detected [20-26].

Recent developments in high throughput sequencing show promise to overcome the limitations in the specificity and dynamic range of microarrays. Next-generation sequencing technology applied to gene expression profiling, known as RNA-Seq, can in principle achieve absolute quantitative measurements of transcript abundance and determine transcript variants with unprecedented resolution [27]. A comparative assessment of global expression profiling through deep sequencing relative to short oligonucleotide microarrays has already been performed 28]. However, RNA-seq has whole transcript coverage and conceptually is more related to tiling arrays or exon arrays and requires far higher coverage. A variation of RNA-Seq known as digital gene expression (DGE) takes advantage of the SAGE methodology principle for sequence based expression profiling, addressing and counting tag sequences next to restriction enzyme sites [29]. DGE is very similar in the sampling approach to long oligonucleotide probe microarray hybridization, given that both techniques take short nucleic acid target sequences to sample expression of longer RNA molecules containing them, and both are 3' biased because they rely on extension of cDNAs from the polyA tail with a oligo-dT primer. Since these are currently the two most cost effective methods for high throughput expression studies, it is of interest to assess the performance of a combination of both methodologies. Microarrays and DGE have already been shown to be comparable in performance [30-35]. In the present study we have used long oligonucleotide microarrays and DGE global cross-validation to present a whole genome perspective of EGF-induced gene transcription and its integration into functional cellular networks. Using the RankProd test applied to multiple platforms, a highly reliable and complete dataset of HeLa specific EGF-dependent regulated genes has been generated defining lists of genes not previously associated to EGF signaling. By applying the recently developed GlobalAncova test for pathway analysis of gene expression profiles, we used this dataset to gain insight into functional aspects and to explore higher order gene regulatory network relationships.

## Results

### Transcriptional profiling of EGF treated cells with multiple oligonucleotide microarray platforms

Global transcriptional profiling can be used to get a snapshot of the state of the cell in a particular condition. To evaluate the genes whose transcription was regulated after 6 h of EGF treatment, treated and untreated control sample pairs were analyzed with long oligonucleotide probe based microarray platforms. In order to generate a well-characterized set of EGF-stimulated and control samples, three independent biological replicate experiments were performed where HeLa cells were serum-starved for 24 h and then stimulated with EGF or left untreated, and verified to show the hallmark signal transduction responses when exposed to EGF (Additional file 1, Figure S1). Three pairs of EGF-stimulated samples and the respective serum starved controls, derived after 6 hours of treatment from each of the same three independent experiments were subsequently analyzed on Agilent, Operon and Illumina microarrays. Normalized and raw data from these experiments are accessible in the GEO database http://www.ncbi.nlm.nih.gov/geo/ under accession number GSE1740.

**Figure 1:**
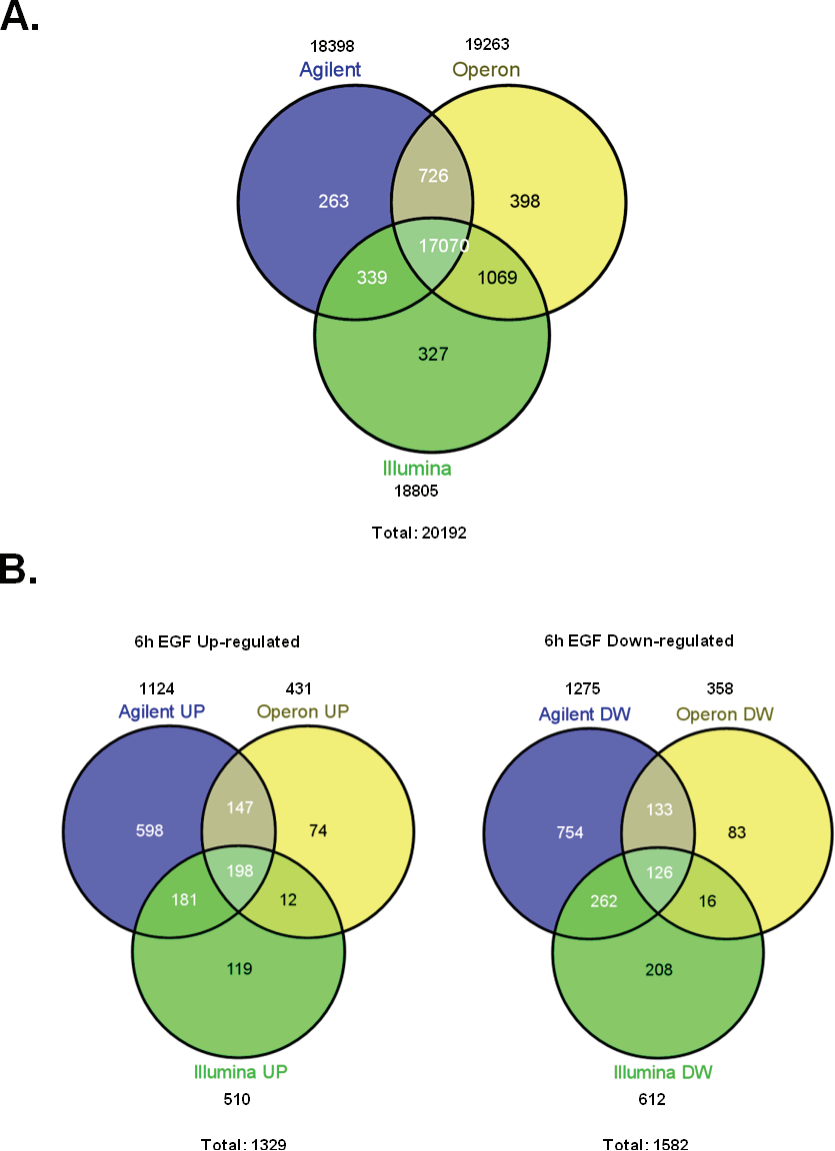
**Microarray interplatform analysis**. (A) Overlap of unique and named genes shared among the 3 microarray platforms used in this study. The pool of 17070 shared genes was used for further cross-platform analysis. The total numbers of genes for each platform and for all platforms combined are indicated. (B) Overlap of significantly regulated genes at 6 h after EGF treatment considering each of the 3 microarray platforms independently.

**Figure 2:**
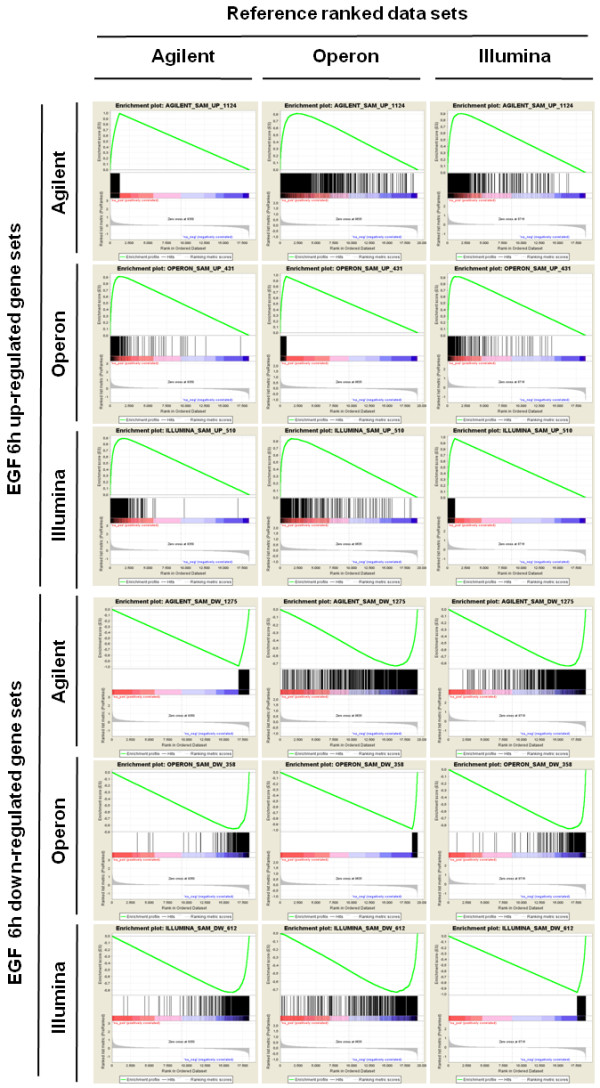
**GSEA analysis on significantly regulated gene sets across microarray platforms**. Profile of the Running ES Score & Positions of Gene Set Members on the Rank Ordered List using 6 h EGF treatment data according to each of the three microarray platforms. In each panel, the vertical black lines indicate the position of each of the genes of the tested gene set in the reference data set (ranked by average of the three respective EGF versus control log2ratios of replicate experiments). The green curve plots the ES (enrichment score), which is the running sum of the weighted enrichment score obtained from GSEA software. Within each queried gene set, the farther the position of a gene to the left (red) implies a higher correlation with EGF up-regulated genes in the reference platform, and the farther to the right (blue) implies a higher correlation with genes down-regulated upon EGF treatment in the reference platform. Studied gene sets correspond to lists of up- or down-regulated genes in each platform at 6 h of EGF treatment. Significantly enriched data sets are defined according to GSEA default settings (p < 0.001 and a false discovery rate (FDR) < 0.25). R.L.M = ranked list metric.

For comparison of results across technologies we focused on RefSeq genes with associated gene symbols. This also simplifies functional analysis given that most genes with known function belong to this group of better annotated genes. Initial comparison between platforms of the rates of change in gene expression expressed as log2ratios using RefSeq remapped probe gene symbols as common identifiers and the median value of all probes for each gene showed a variable degree of correlation. These platforms have 17,070 RefSeq genes in common (Figure 1A). The first exploration of the data trying to find shared regulated genes, showed a strikingly low degree of overlap between the lists of most significantly regulated genes, when determined by applying an absolute fold change cut-off of 1.2 and setting a false discovery rate at 5% with significance analysis of microarrays (SAM) (Figure 1B**; **Additional file 2, Table S1). The reduced overlap observed is consistent with previous reports of small intersection between lists in similar experimental designs [21,26,36]. We then used gene set enrichment analysis as implemented in the GSEA tool [37] (which takes into account the entire distribution of log2ratios) to increase the power of the comparison of the results of all three platforms [36]. Our GSEA analysis showed a highly significant agreement between all three platforms, since each gene set identified by any of the three platforms was found to be asymmetrically distributed within the remaining rank ordered differential gene expression datasets (GSEA FDR q-value = 0 for all comparisons) (Figure 2**; **Additional file 3, Table S2). This result strongly argues in favor of all platforms being able to detect the same underlying transcriptional response behavior, while differences among individual gene measurements make it more difficult to detect these common properties when focusing only on the intersection between the top significant gene lists from the individual platforms.

Upon comparing different datasets, t-test based methods, such as SAM, are less sensitive and more prone to give false positives than rank product-based tests [38]. In fact this may explain the low overlap obtained using SAM derived gene lists. After proving with GSEA that the datasets were truly comparable, the RankProd test was applied to determine a statistically significant gene list based on multiple platforms [39]. Given that there are quite a few instances where data are discrepant between platforms, we used this test to identify the most likely result based on objective statistical criteria, coming up with 656 upregulated and 596 downregulated genes in response to EGF based on 3 independent microarray platforms with an absolute median fold change larger than 1.2 and an adjusted p-value of the RankProd test below 0.05 (Additional file 4, Table S3).

Gross EGF-specific expression cell type specific biases attributable to the HeLa molecular karyotype were excluded by correlating expression data with copy number using array based competitive genomic hybridization (Data not shown).

### Digital expression profiling by high throughput tag sequencing

**Figure 3:**
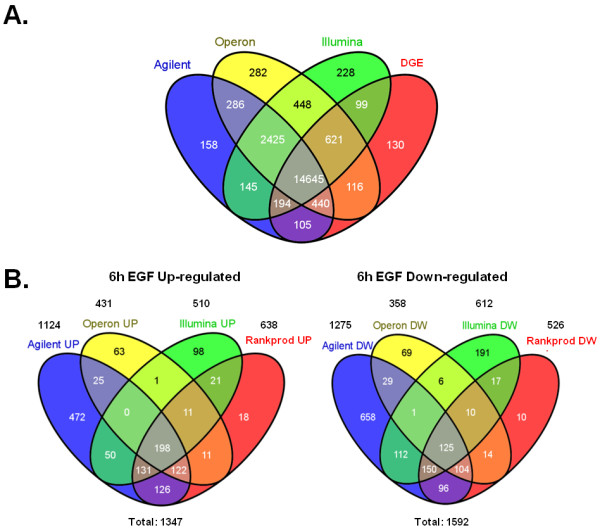
**Microarray versus DGE analysis**. (A) Overlap of unique and named genes shared among the 3 microarray platforms and genes detected by DGE. The pool of 14645 shared genes was used for further cross-platform analysis. The total numbers of genes for each platform and for all platforms combined are indicated. (B) Overlap of significantly regulated genes considering the 3 microarray platforms at 6 h after EGF treatment and the genes found regulated after assessing significance by grouping microarray and DGE data in a RankProd analysis. Left panels show up-regulated genes and right panels show down-regulated genes.

**Figure 4:**
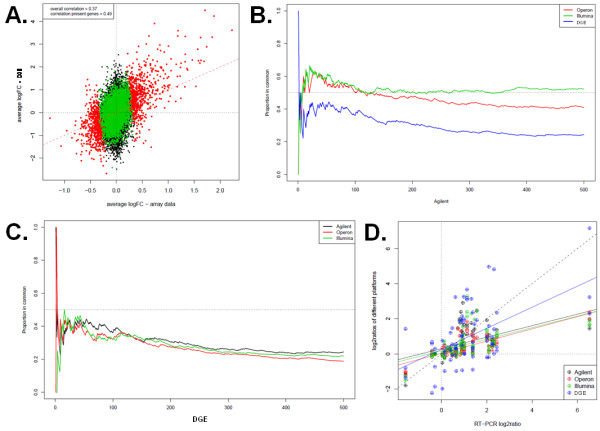
**Correlation between microarrays and Illumina GA-I sequencing**. (A) Comparison of estimated log2ratios from DGE (*Y*-axis) and the mean of all microarray platforms (*X*-axis). We consider only genes that were interrogated using all platforms and genes with a mean number of counts across lanes greater than 0. Genes with counts greater than 32 reads (colored red or green) or less than (black) 32 reads in at least one sample are shown. (Red dots) Genes called differentially expressed based on DGE data at an 10% FDR by RankProd. (Green dots) Genes not called as differentially expressed but above 32 counts. (Inset box) Correlation between technologies is higher when considering genes above the 32 count detection level (0.57) than when all genes are included (0.49). (B-C) Concordance at the top (CAT) plots of the different platforms with the 500 top genes from a reference platform, shown for Agilent in (B) and DGE in (C). See inset box for color codes identifying each platforms compared to the remaining platform used as reference. (D) Correlation plots with regression lines between log2ratios of the five high content platforms measurements (Y-axis) and quantitative real time PCR results using SYBR green assays (X-axis), based on measurements for 21 genes at the 6 h time point (see Additional file 2, Table S1).

The final gene lists obtained from microarray data analyses are only a partial representation of the transcriptome due to the fact that the genes surveyed are constrained to the probes present in each array, and because the overlap in gene coverage and in differential gene expression detection between platforms is incomplete. Ideally, it would be desirable to have a detailed and comprehensive gene list of EGF-dependent genes. The only way to extend the validation without being limited by the probe content of each platform is to use an open technique. For this reason we used the DGE methodology developed by Illumina which is based on the SAGE principle but up-scaled on the Genome Analyzer I (GA-I) next generation sequencing platform [30-35]. We re-analyzed aliquots of total RNA from the exact same three replicate experiments that had been tested on microarrays: serum-starved and EGF-treated for 6 h. On average, 9 × 10E6 raw sequences were obtained per sample, which after running the analysis pipeline allowed us to monitor the expression of 4.9 × 10E6 unambiguously matching tags, corresponding to 16,350 different genes (as determined from RefSeq unique gene symbols) (Table 1; Additional file 5, Table S4). This number has been considered sufficient by others to achieve over 90% coverage of the transcriptome, with as high or higher sensitivity than short oligonucleotide probe microarrays [33]. 16,220 of the 17,070 genes represented in every microarray platform could be detected through DGE. 3,972 genes represented in either of the 3 microarray platforms had no detectable measure by DGE in any of the three biological replicates, whereas 130 detected tag sequencing targets had not been addressed by any of the microarray platforms (Figure 3A). Neither SAM nor RankProd statistical analysis of differential gene expression by DGE gave any significant genes after multiple testing correction. A general comparison between microarrays versus deep sequencing showed better correlation among genes that had 32 or more counts in their tag sequences (Figure 4A). Following, we used CAT ('concordance at the top') plots [40] representing the changes among the proportions of genes shared between gene lists ranked by fold change as a measure of the concordance between each of the different microarray platforms and DGE compared to our reference microarray platform (Agilent, Figure 4B). We then compared all microarrays to the DGE dataset (DGE, Figure 4C), showing that there is a significant degree of agreement between the three alternative commercial array platforms and DGE (Figures 4B and 4C). These plots show that the concordance is highest between the top 100 genes and that, as we increase the list size, the proportion of genes shared among lists stabilizes around 45-50% between microarray platforms and around 30% between microarrays and DGE. In part this is explained by the fact that EGF regulates many genes and the fold changes detected by each platform are correlated but the exact ranking can vary a lot given the large number of genes affected. In agreement with this, gene set enrichment analysis showed a significant correlation between the 3 microarray platforms and DGE (Data not shown).

**Table 1:** Deep tag sequencing statistics

	**RefSeq**	**GeneID**	**Genscan**	**RNAgenes**	**total**	**% of** total	**% of** **unamb**.	**Averages/** run
total reads (6 runs)	54072498	14042145	6265092	5244975	54072498	100.00%		9012083.00

unambiguous (6 runs)	24940641	3665152	779972	263786	29649551	54.83%	100.00%	4941591.83

0 mismatches	18084026	286158	5470	95713	18471367		62.30%	3078561.17

1 mismatch	3913853	1961500	102902	87180	6065435		20.46%	1010905.83

2 mismatches	2942762	1417494	671600	80893	5112749		17.24%	852124.83

ambiguous (6 runs)	15083422	4111901	240145	410907	19846375	36.70%		3307729.17

no matches (6 runs)	14042145	6265092	5244975	4570282	4570282	8.45%		761713.67

Summary statistics of the mapping of reads generated by the DGE pipeline.

**Figure 5:**
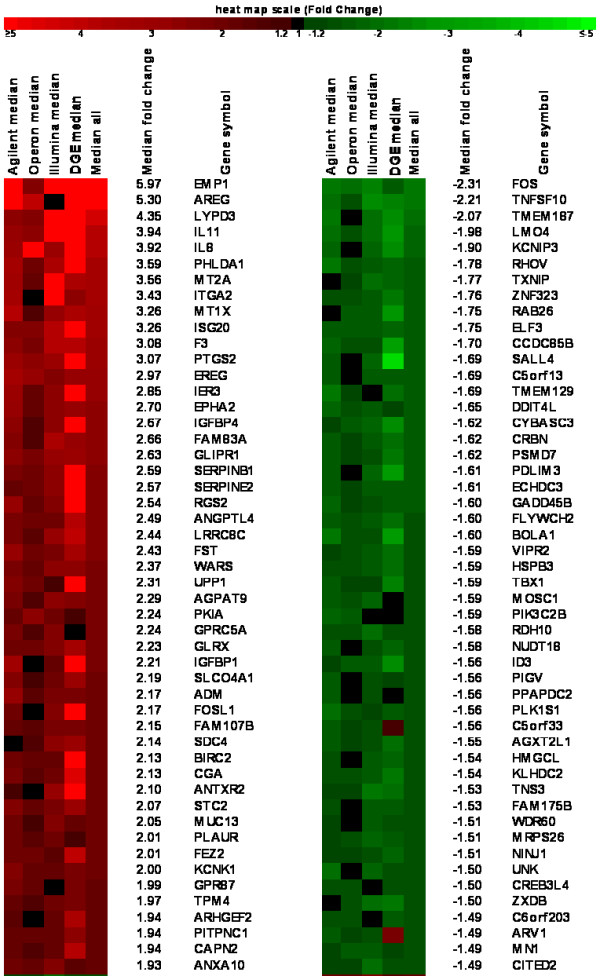
**Top regulated genes derived from meta-analysis**. RankProd analysis of the combination of microarray and Illumina GA-I ultrasequencing data sets. Heatmap of the top 50 up and down-regulated genes detected in all four platforms ordered by Median Fold Change (all have RankProd adjusted p-values < 0.0001). IL11, IL8, PLAUR, ANXA10 and FOS were validated by RT-qPCR showing concordant results (See Additional file 2, Table S1). The full RankProd matrix from these experiments is accessible in Additional file 6, Table S5. The list of all 1164 significantly regulated genes (median |FC| > 1.2 and RankProd q-value < 0.05) is given in Additional file 7, Table S6.

The RankProd analysis to integrate microarray and DGE data (Figure 3B**; **Additional files 6 and 7, Tables S5 and S6) allowed us to define a list of 638 upregulated and 526 downregulated RefSeq genes in response to EGF at 6 h (RankProd test adjusted p-value: p < 0.05, median absolute fold change of all measurements: |FC|>1.2; genes represented in any of the four platforms) (Figure 5**; **Additional file 7, Table S6). The number of genes found significant by RankProd when combining microarray and sequencing data together is slightly lower than that found significant by microarray only. This implies that the vast majority of genes cross-validated by microarrays turned out to give concordant results by DGE. Even though the total number of genes was reduced, DGE added 28 new genes not detected by microarrays to the RankProd-significant regulated gene list (18 up and 10 down).

For a small collection of genes, independent experimental validation was performed using a SYBR green based RT-qPCR assay on the exact same samples used in microarray and ultrasequencing experiments. Some of them were further validated in additional samples in a time course experiment. Most of the genes analyzed by RT-qPCR showed concordant results with all technologies used in this study (Additional file 8, Figure S2). In order to assess linearity in each genomic analysis assay, we plotted the log2ratio values of the subset of 28 genes validated by RT-PCR (Figure 4D) and found that DGE approximated best the fold change detected by RT-PCR. It is noteworthy that while all microarray platforms had similar specificity and sensitivity in detecting changes in gene expression, DGE had more false positives, particularly among genes represented by a low number of tags (Additional file 9, Figure S3).

We then used multiple approaches for the functional analysis of the genes found regulated by EGF including GO enrichment analysis (with EASE), gene set enrichment analysis (with GSEA), literature based network inference (with Ingenuity) and a general test applied to KEGG pathways (with GlobalAncova). Interestingly with GSEA using literature defined genesets (c2 MSigDB subset) we were able to recover with very high significance those defined by Amit et al [11] as response signatures to EGF in HeLa cells at 4 (FDR and 8 hours, the time points that are closest to ours; data not shown). This further supports that in our hands the system behaved as it has been described by others.

We applied these same tools to the reduced dataset including the overlap but also to all genes (including those that were only represented in some of the platforms). Using this approach, we detected once again the classical EGF pathway plus a few other related functions such as genes known to modulate EGF signaling, non-EGF EGFR agonists, known EGF-responsive transcription factors, components of ERBB receptor-associated trafficking and EGFR interacting proteins (Additional file 10, Figure S4).

We also analyzed an extended dataset including, in addition to the genes shared in common, those only represented by a single platform or a subset of all platforms. One of the most significant hits found when using the inclusive dataset was the copper/cadmium metallothionein metal ion homeostasis function, which includes a few of the most differentially expressed genes 6 hours after EGF treatment and although individual platform analysis uncovered this pathway only in Agilent arrays (Additional file 11, Figure S5A) we validated these observations using RT-qPCR for 6 of the human metallothionein family members. Results indicate that all metallothionein genes studied but MT1F are up-regulated after EGF treatment (Additional file 11, Figure S5B). This result went unnoticed in an EGF time course treatment of HeLa cells [11] performed on Affymetrix arrays also showing consistent and progressive up-regulation of MT1E, MT1G, MT1F, MT1H, MT1M, MT1X, MT1P2, MT2A (MT1A and MT1B were not represented in the Affymetrix U133A platform used in this other study) (Additional file 11, Figure S5C). This may be indicative of a novel function of EGF which may be to activate oxidative stress protection and metal ion homeostasis through up-regulation of most metallothionein genes. This example shows that there may be inconsistencies in probe design that can lead to results that are not reproducible in other platforms and highlights the risk of picking up results that are platform biased when relying on just a single platform and the fact that there is many hidden information in already published datasets that can be uncovered using the approaches described in the present work.

### EGF-dependent functional networks

**Figure 6:**
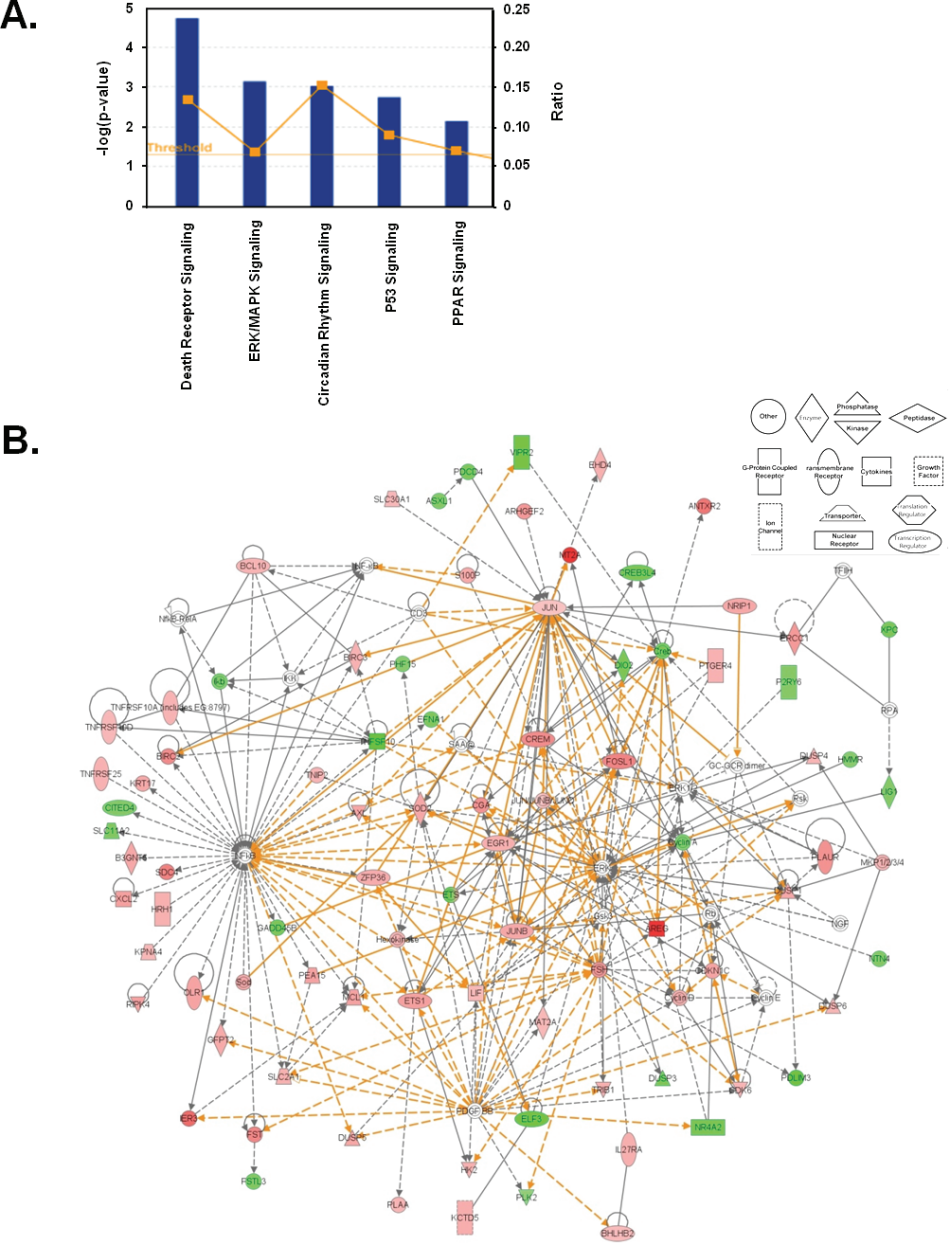
**Significant pathways and interactions among EGF-regulated geneset**. (A) Core functional analysis of EGF-regulated genes derived from the RankProd test clustering around canonical pathways performed using the Ingenuity Pathway Analysis software. (B) Pathway analysis based on the Ingenuity Pathway Knowledge base. The two best ranked networks holding EGF-regulated genes derived from the RankProd test were merged showing a unique network. Up-regulated genes are indicated in red and down-regulated genes in green. The shape of the node denotes the main function of the protein encoded by the gene (see boxed inset). Continuous lines indicate interaction between the products of the genes; dashed lines indicate an indirect interaction; lines with an arrow indicate that the source gene "acts on" the target gene. Regulated genes are shown as grey boxes and non-regulated but associated with the regulation of some of these genes are shown as white nodes. Orange lines indicate new gene relationships appearing after merging different networks.

To further investigate the global expression response to EGF treatment as well as to study the interaction between individual regulated genes and how they have a coordinated role in specific signaling pathways, we used the IPA (Ingenuity Pathway Analysis) software, using the 1146 genes obtained by RankProd testing (adjusted p-value: p < 0.05, median absolute fold change of all measurements: |FC| > 1.2). Among the top molecular and cellular categories, we observed the presence of the most common functions related to EGF signaling such as cell death, cell growth and proliferation [1], being cancer the top disease. In all cases, the biological functions identified have a very high overlap in gene content. This is in agreement with the top regulated canonical pathways described by IPA which are: cell death, cancer, and cellular growth and proliferation. (Figure 6A and Table 1).

The top ranked networks identified by IPA are associated with cell death and survival, cellular proliferation, and tissue development and function (Table 2). Networks 1 and 2 (Additional file 12, Figure S6) consist of genes most of which interact directly with NF-kB and ERK1/2. Upon EGF stimulation both proteins are activated, NF-KB is activated through the AKT pathway and ERK1/2 is activated by MEK phosphorylation, being the expression of these two genes themselves not regulated at the transcriptional level upon EGF treatment [1].These two highest scoring networks showed a high degree of interconnectivity as shown through merging (Figure 6B).

**Table 2:** Functional analysis of differentially expressed EGF responsive genes.

TOP NETWORKS		
Associated Network Functions	Focus Molecules	Score
1. Cell Death, Embryonic Development, Renal and Urological Disease	28	45
2. Amino Acid Metabolism, Post-Translational Modification, Small Molecule Biochemistry	26	40
3. Cell Cycle, Cancer, Cardiovascular System Development and Function	24	35
4. Cellular Growth and Proliferation, Hematological System and Connective Tissue Development and Function	24	35
5. Cellular Movement, Cellular Assembly and Organization, Cell-to-Cell Signaling and Interaction	24	35

		
**TOP BIOLOGICAL FUNCTIONS**		
		
**Molecular and Cellular Functions**	**p-value**	**Molecules**

1. Cell Death	7.41e^-19^-6.45e^-04^	145
2. Cell Growth and Proliferation	1.77e^-16^-6.15e^-04^	160
3. Cellular Movement	3.16e^-12^-6.50e^-04^	101
4. Cellular Development	9.85e^-11^-6.28e^-04^	115
5. Cell Cycle	1.23e^-10^-6.63e^-04^	75

		
**Diseases and Disorders**	**p-value**	**Molecules**

1. Cancer	1.82e^-17^-6.63e^-04^	193
2. Reproductive System Disease	5.14e^-15^-6.57e^-04^	98
3. Immunological Disease	1.09e^-10^-6.63e^-04^	70
4. Dermatological Disease and Conditions	1.59e^-08^-3.33e^-04^	61
5. Inflammatory Disease	1.59e^-08^-5.61e^-04^	58

List of Ingenuity Networks and Biological Functions generated by mapping the 1164 focus molecules that were differentially expressed during EGF treatment according to RankProd.

**Figure 7:**
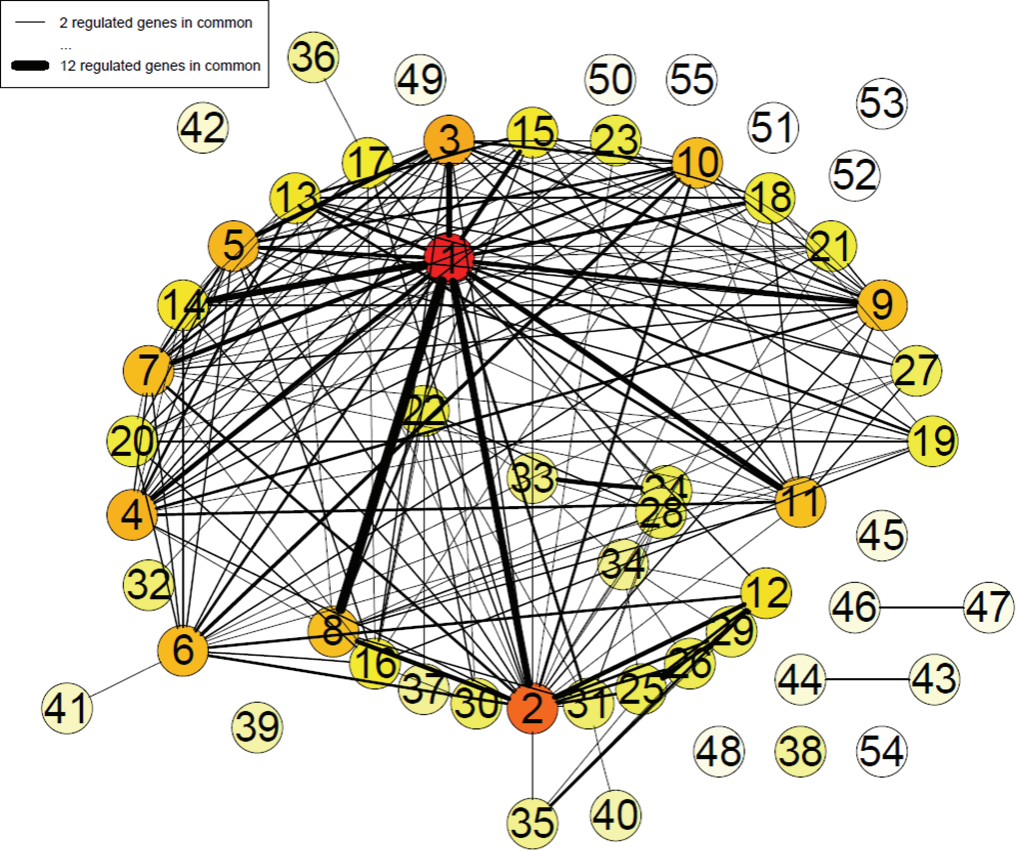
**Higher order network of interactions among EGF-regulated genes**. Network of genomic interactions among EGF-regulated pathways (Holm-adjusted p-value < 0.01) as defined by GlobalAncova using KEGG database functional annotation. Nodes (pathways) that have at least two regulated genes (as defined by RankProd analysis) in common with other pathways are connected by continuous lines to these other pathways. The strength of each pathway interconnection (i.e. the number of shared regulated genes) is expressed by the width of the continuous lines connecting the two nodes. The node color indicates the interconnectivity of the nodes ranging from no connection to any other pathway (white) to many connections with other pathways (red). Numbers define KEGG categories as listed in Table 3.

We asked ourselves if this interconnectivity between networks would allow us to model a higher order network in these interactions. In order to measure pathway interconnectivity, the GlobalAncova method was applied on the classical pathways (as defined in the KEGG database). In this approach a global regulation score is computed for each pathway taking into account the expression values of all the genes belonging to it. Again, this analysis indicated that many of the regulated pathways are not independent since they share a large number of genes (Figure 7). As expected, many pathways related to cell growth and proliferation, cell death and cell cycle are represented. Many of the most significant pathways belonged to the signal transduction class and contained the hub proteins central to the networks found significant by IPA analysis. In addition, among disease related pathways, the top regulated ones were mostly related to cancer, being "Pathways in cancer" the top one with a total of 31 genes and 131 connections (Table 3**; **Additional file 13, Table S7 for full GlobalAncova analysis).

**Table 3:** Functional analysis of EGF responsive pathways.

	ID	name	genes	regulated genes	connections
1	05200	Pathways in cancer	265	31	139
2	04510	Focal adhesion	156	17	97
3	04660	T cell receptor signaling pathway	80	7	69
4	05215	Prostate cancer	74	7	65
5	04662	B cell receptor signaling pathway	59	6	63
6	04010	MAPK signaling pathway	203	25	62
7	05210	Colorectal cancer	55	6	61
8	05222	Small cell lung cancer	76	12	60
9	04012	ErbB signaling pathway	74	9	60
10	04722	Neurotrophin signaling pathway	104	9	60
11	05220	Chronic myeloid leukemia	64	7	59
12	04810	Regulation of actin cytoskeleton	163	14	46
13	05214	Glioma	55	5	44
14	05211	Renal cell carcinoma	58	9	44
15	05223	Non-small cell lung cancer	51	6	42
16	05142	Chagas disease	71	5	41
17	04310	Wnt signaling pathway	118	7	41
18	05219	Bladder cancer	35	5	37
19	05140	Leishmaniasis	42	4	36
20	04620	Toll-like receptor signaling pathway	59	4	36
21	04912	GnRH signaling pathway	70	6	35
22	04210	Apoptosis	74	8	32
23	04916	Melanogenesis	76	3	30
24	04630	Jak-STAT signaling pathway	94	12	27
25	05410	Hypertrophic cardiomyopathy (HCM)	60	7	27
26	05414	Dilated cardiomyopathy	65	7	27
27	04115	p53 signaling pathway	58	11	25
28	04621	NOD-like receptor signaling pathway	40	6	25
29	05412	Arrythmogenic right ventricular cardiomyopathy (ARVC)	52	6	25
30	04920	Adipocytokine signaling pathway	55	6	24
31	04120	Ubiquitin mediated proteolysis	120	15	22
32	04370	VEGF signaling pathway	57	3	21
33	04060	Cytokine-cytokine receptor interaction	132	14	18
34	04640	Hematopoietic cell lineage	43	7	16
35	04530	Tight junction	97	7	16
36	04340	Hedgehog signaling pathway	39	3	15
37	04622	RIG-I-like receptor signaling pathway	46	4	15
38	04360	Axon guidance	105	9	14
39	05217	Basal cell carcinoma	41	1	12
40	04144	Endocytosis	162	13	11
41	05014	Amyotrophic lateral sclerosis (ALS)	41	3	8
42	04350	TGF-beta signaling pathway	69	6	6
43	00561	Glycerolipid metabolism	38	4	5
44	00564	Glycerophospholipid metabolism	59	4	5
45	00600	Sphingolipid metabolism	27	4	4
46	04070	Phosphatidylinositol signaling system	66	4	4
47	00562	Inositol phosphate metabolism	50	3	3
48	00565	Ether lipid metabolism	20	1	3
49	05020	Prion diseases	22	3	3
50	04710	Circadian rhythm - mammal	20	4	2
51	00601	Glycosphingolipid biosysnthesis - lacto and neolacto series	21	2	0
52	00532	Glycosaminoglycan biosynthesis - chondroitin sulphate	17	2	0
53	00760	Nicotinate and nicotinamide metabolism	17	4	0
54	00750	Vitamin B6 metabolism	5	1	0
55	00790	Folate biosynthesis	9	0	0

List of GlobalAncova derived differentially expressed KEGG functions upon EGF treatment indicating the total number of genes, the number of regulated genes and the number of connections (shared regulated genes) to other pathways. The number identifying each KEGG category are the same used for the nodes in the graph on Figure 7.

## Discussion

### EGF response gene signatures and higher order network inference

Most of functional analyses performed on microarray datasets are usually applied to data that were derived from a single microarray platform, where often only the expression of a few genes has been validated experimentally by alternative methods, usually RT-qPCR. In such cases, it is assumed that the measures of hundreds to thousands of targets on an array are 'true' measurements. As has been noted in many studies, and as we show in the present study, a significant percentage of probes on any single platform can show discrepancies with results derived from probes for the same target genes in different platforms or obtained with an alternative technology. The MAQC landmark multi site study focused on the ability to capture global differences by different platforms and in intra platform reproducibility and sensitivity, but did not address how to integrate data derived from different platforms [41,42].

We have focused on generating gene lists extensively cross-validated by different methodologies on the same set of samples to ask a biologically relevant question at the same time. We define a list of genes that has shown consistent regulation by EGF in three different microarray platforms as well as by DGE using next generation sequencing of short tags. By using this high content cross-validation based approach we are providing a large and reliable dataset capturing the EGF-dependent transcriptome in HeLa cells. This expands the previous knowledge of this process, not only providing a robust list including previously known target genes, but also expanding it with a fair number of genes under EGF-regulation that had not previously been associated to EGF. In addition, we are able to define a large EGF dependent gene network using the high interconnectivity observed among the minor pathways regulated by EGF. The role of EGF/EGFR dynamic interaction networks has been studied recently with either computational approaches [43], or by integration of molecular profiling, database and literature mining, mechanistic modeling, and cell culture experiments, demonstrating that EGF (among other growth factors) plays an important role in communication networks regulating blood stem cell fate decisions [44].

The 6 h EGF time point was chosen because of the high amount of transcriptional regulation which includes some well established sets of targets (that allowed us to use known targets as positive controls) and largely unknown regulatory mechanisms. The 6 h EGF time point captures the steps following initial EGF pathway activation of early response transcription factors (JUN, FOS, MYC, EGR3), the negative feedback regulation mediated by their post-transcriptional targets (DUSP family of dual specificity phosphatases), the increase in delayed response transcription factor activation (and downfall of the early response genes) and the activation of the regulatory mechanisms that will determine the cell fate as either apoptosis (BCL10; BIRC2/3; GADD45A/B; TNFR family receptors) or continued proliferation and survival (cyclins, cycling dependent kinase inhibitors, growth factors, cytokines).

Upon thorough functional analysis of microarray and ultrasequencing data focused on the 6 h time point, we were able to detect cell death, cell growth and proliferation, cellular movement and development responses to EGF stimulation. These are the functional categories appearing as significantly overrepresented using a range of methods and tools with the set of genes that come out significant in the multi-platform RankProd analysis and that are present in all platforms. It allowed us to confirm that our system is behaving as would be predicted from prior knowledge. Given the robust nature of our data, at the same time we can infer network relationships based on true changes in gene expression.

Networks result from interconnections between signaling pathways. Such interconnections occur because the same signaling component is capable of receiving signals from multiple inputs or it can distribute its signal to different pathways. We have used the genes involved in several networks as interconnecting genes to build supra-pathway structures. A major limitation was found in the fact that current versions of pathway databases are not completely up to date. Many of the genes not currently included in the classical pathways could be added upon close inspection of the literature. While this does not appear to affect the major pathways involved, in any case, it reinforces them. From the 44 statistically overrepresented pathways only 8 of them have no connections with any other pathway. Keeping in mind the limitations of the KEGG database we can conclude that there is extensive interconnectivity between EGF-regulated genes in our dataset.

The EGF signaling network includes survival pathways and interacts at many levels with the apoptotic signaling network, being able to influence on the apoptotic potential of cells modulating and regulating the balance between survival and death. A thorough understanding of the genes that can be modulated by EGF and all the interactions is critical for success on rationally designed cancer treatments. We observed a clear cross-talk between the EGF anti-apoptotic pathways and the apoptotic pathways. EGF signaling leads to the up-regulation of anti-apoptotic proteins, blocking the extrinsic (death receptors) and intrinsic (mitochondrial) pathways or inactivating of pro-apoptotic proteins. Interestingly, specific cancer pathways are highly represented and interconnected among themselves and with signaling pathways involved in cancer including Wnt, TGF-beta, MAPK, p53 and other.

Hubs are proteins interacting with many partners and its study is becoming of great interest. Essential proteins tend to belong to biological processes that are densely interconnected and are more likely to be hubs [45]. Interestingly, in our IPA analysis we find three main hubs linking many regulated gene networks: ERK/MAPK, NFKB and PI3K. While the mRNA levels of the genes encoding for the hub proteins themselves are not affected by EGF, we can detect strong changes in many of the genes directly connecting to them and a high interconnectivity among regulated genes pertaining to each hub's own network.

### Novel gene functions regulated by EGF

As pointed out, most of the genes found to be regulated by EGF were related to already known functions such as cell cycle, differentiation or apoptosis, which were detected as significant even when looking at the most conservative gene lists obtained by combining all platforms together.

Being less conservative, one can attempt to look at the global picture of EGF response by not only looking at the common intersection of genes represented in all platforms, but at the union of all the identifiers. Using this approach to try to uncover novel functionalities, it was interesting to detect regulation of additional genes in categories described to modulate EGF signaling (such as DUSP dual specificity phosphatases, SOCS suppressors of cytokine signaling, ERRFI1 and LRIG) [46], most non-EGF EGFR agonists (TGF-alpha, epiregulin, amphiregulin, HB-EGF and epigen) and the CXCL1/2/3 cytokines, which interestingly are cytogenetically linked to a cluster of EGF family members on human 4q13.3 along with IL8 and are found to be co-regulated. In addition, there are changes found in mRNA levels of transcription factors of the early response and delayed early response class [11], some of the components of ERBB receptor endocytosis and intracellular trafficking complexes [47] and EGFR interacting proteins [48]. This observation supports the existence of tight feedback mechanisms 6 h after exposure to the EGF ligand. The purpose of these would be to shutdown EGF dependent signaling through transcriptional up-regulation of inhibitors, in agreement with the results of Amit et al [11], along with the parallel compensatory up-regulation of other growth factors that act through the same ERBB receptor family.

In the attempt to uncover additional new functions on the conserved dataset and extended datasets using several approaches, we detected a significant overrepresentation of metallothionein genes regulated 6 hours after EGF treatment, both as the cadmium and copper ion homeostasis functional category and the 16q13 cytogenetic band by enrichment analysis. Metallothioneins are known to be regulated by many stimuli such as oxidative stress, metal ions and glucocorticoids. Indeed, the putative role of metallothioneins in carcinogenesis has been proposed recently [49].Our work highlights their regulation by EGF, not yet reported to date.

It remains to be seen whether this regulation is a direct result of transcriptional activation by EGF primary targets. Indeed, the presence of AP-1 elements in the metallothionein promoter would provide a likely mechanism of activation by EGF-dependent early response genes.

### Contribution to cross-platform validation

Often, studies using multiple platforms have been carried out on highly heterogeneous samples with very divergent expression profiles and on a limited number of platforms, focusing on the common top regulated genes, excluding the non-overlapping, and therefore missing potentially relevant regulated genes [50]. Because the measures of gene expression themselves cannot be directly compared among different platforms [51], we found that the use of rank comparison tests can serve as a way to increase the number of regulated genes given that similarities in gene regulation are made less dependent on the magnitude of change or the gene expression measures themselves. Our datasets reveal overall agreement for many genes surveyed, yet there are quite a large number of probes that give discrepant results. We performed an outlier analysis and were able to detect the highest degree of disagreement (comparing each microarray platform to the rest) in Operon, followed by Illumina and Agilent (Data not shown). In our metallothionein example, it was evident that the major differences came from the subset of the genome represented on each platform. It is worth to note that effectively remapping of all the probes in different platforms indicated that there is a considerable number of probes that do not match RefSeq transcripts (data not shown). Stringent reanalysis of published data using these platforms should take this into consideration. In addition, we find that many probes have ambiguous matches in other transcripts, indicating them as likely mediators of cross-hybridization artifacts.

### Assessment of DGE performance compared to microarrays

Our basic analysis of the data generated in this work indicates that DGE methodology is quite sensitive but noisier than microarrays themselves. Previous reports that have shown improved performance of DGE over microarrays have made comparisons against short oligonucleotide probe platforms such as Affymetrix, and have used larger numbers of reads effectively increasing dynamic range and sensitivity at a higher cost per sample [33]. There appear to be many challenges to be solved to correct for this noise: first, there are many more differences found in the number of tags for specific genes in biological replicates of the same conditions than would be expected from our microarray experiments; second, the normalization applied, referring to the total number of counts, may not be the best method (as with microarray data, more sophisticated methods may be required). Our end result was the finding of higher fold changes accompanied by poorer reproducibility among biological replicates in DGE data relative to microarrays. This, for the moment, makes this DGE method not optimal to be taken as golden standard, pointing to the need to improve the technology or have some other means of experimental cross-validation as we reported in this study. In this sense, while adding RT-qPCR data on a few genes may still be sufficient for publication under current standards, our microarray experiments would support that global validation to confirm larger sets of genes may be more appropriate, especially when gene lists derived from these studies are exploited for data integration and systems modeling.

One unexpected finding was the considerable number of genes not detected by DGE that were detected using microarrays. This absence of tag detection could in part be explained by the lack of restriction sites that would prevent these sequences from being represented in the libraries generated in the DGE assay. Consistent with this possibility 1.5% of the tags from DGE for which no log2ratio could be computed in any of the three biological replicates due to absence or too low number of tags, actually lacked DpnII sites.

Most tags only detected by DGE (99.73%, corresponding to the 1488 transcripts), had DpnII restriction sites mapped in their RefSeq database sequence. These are transcripts not represented in any of the three microarray platforms, but this fact does not necessarily argue in favor of DGE being more sensitive.

Our ability to compare up to four different platforms allows us to attempt to provide tools for identifying suboptimal probes in each of several commonly used long oligonucleotide microarray platforms. We have generated extensively cross-validated benchmark datasets that can be used to fine tune analysis algorithms both for long oligonucleotide microarray and short-read, tag-based gene expression data.

## Conclusions

In our analysis using three long oligonucleotide microarrays platforms and digital gene expression we explored in depth the transcriptional response to the well-established EGF-dependent signal transduction pathway.

Knowing that there are biases in genomic studies that are platform dependent, our study attempted to get around this limitation to increase the confidence in the transcriptome changes detected, in order to allow more reliable analyses at the functional genomics level and to try to infer more robust networks of co-regulated genes which may benefit further genomic studies with the obtained datasets.

Performance comparison between microarray and next generation based digital expression profiling suggests that the two methodologies combined may survey the transcriptome in a better way than each on its own, and therefore generate more reliable datasets and uncovering additional new functions. Ongoing improvements in data quality and increased output of Illumina sequencing technology make it possible to achieve higher read depth and less noise at a reduced cost, which would make DGE today even more attractive as a tool for studying gene expression. Even though currently RNA-seq is the most comprehensive methodological approach to assess transcript abundance and complexity, DGE is conceptually more comparable to microarrays. Therefore, we believe DGE is the ideal complementary technique for global cross-validation of long oligonucleotide microarray data applied to quantitative expression profiling.

Indeed, this approach, where data from both technologies is integrated through RankProd analysis, is capable of detecting new genes that may previously have gone unnoticed acting downstream of EGF and that had not been described at a global level before. For the metallothionein family this has relevance for cancer studies since these are genes often deregulated in cancer and that may be important in relationship to cancer resistance to chemotherapy. We propose that cross-validation technologies may be exported to the desired paradigm with the same advantages as the described in this paper.

## Methods

### Reagents & Antibodies

EGF from murine submaxillary gland and anti Tubulin (1:10000) were purchased from Sigma. Anti p-ERK1/2 (1:2000), Anti p-p90rsk (1:2000), anti p-EGFR (1:1000), anti p27 Kip1 (1:1000) and anti p-CREB (1:2000) were from Cell Signaling. Anti Cyclin D1 and anti cyclin E were from Santa Cruz. U0126 and AG1478 were from Calbiochem.

### Cell Culture and Sample preparation

HeLa cells were cultured at 37°C in a 95/5 Air/CO_2 _water saturated atmosphere in Dulbecco's modified Eagle's medium (DMEM) containing 10% heat inactivated fetal bovine serum (FBS), 2 mM L-glutamine and 100 U/ml Penicillin/streptomycin. For treatments, the cells were transferred to 60 mm dishes and, after 48 h, starved for 24 h in DMEM containing 2% FBS. The cells were incubated (if indicated) with the protein kinase inhibitors U0126 (10 μM) or AG1478 (10 μM) for 30 min, and then stimulated with EGF (150 ng/ml) for the indicated times. Cells were harvested, washed twice with cold phosphate-buffered saline and lysed with either 2 × Laemmli sample buffer (Sigma), for protein extraction, or RNeasy RLT lysis buffer (Qiagen), for total RNA extraction.

Total RNA was quantified with a NanoDrop ND-1000 spectrophotometer followed by quality assessment with the 2100 Bioanalyzer (Agilent Technologies) according to the manufacturer's instructions. Acceptable quality values were in the 1.8-2.2 range for A260/A280 ratios, >0.9 for rRNA ratio (28S/18S) and >8.0 for RIN (RNA Integrity Number).

### Western Blot

For Western blotting 50 μg of cell extracts from HeLa cells were subjected to 8-10% SDS-PAGE. Gels were transferred onto PVDF membranes and processed for specific immunodetection by ECL using the antibodies at the dilutions indicated above.

### RT-qPCR

Quantitative real time PCR was performed on two sets of genes. The first set was validated on the original three biological replicate experiments analyzed by microarrays and DGE (set 1: DUSP1, DUSP6, IL8, CCND1, CCNE2, MYC, FOS, CDKN1A, CDKN1B, CDKN1C, MAP3K6, IL11, EGFR, AURKC, E2F1, TGFA, CEBPD) and the second set on three independent biological replicates (set 2: MT1E, MT1F, MT1G, MT1H, MT1X, MT2A). Total RNA was extracted from HeLa cells, for set 1, with mirVana isolation kit (Ambion) and, for set 2, with miRNeasy Mini kit (Qiagen) following the respective manufacturer's instructions. Purified RNAs were treated with RNase-free DNAse (DNA-free, Ambion) and reverse-transcribed, for set 1, with Superscript II (Invitrogen) and, for set 2, Omniscript (Qiagen) to generate the corresponding cDNAs that served as PCR templates for mRNA quantification. Primers used in this study for RT-qPCR validation can be found on Additional file 14, Table S8.

PCR amplification and detection were performed with the ROCHE LightCycler 480 detector, using 2 × SYBR GREEN Master Mix (Roche) as reagent and oligonucleotide primers (0.25 uM or 0.3 μM of each primer, for set 1 and set 2 respectively) following the manufacturer's instructions. The reaction profile had a denaturation-activation cycle (95°C for 10 min) followed by 40 cycles of denaturation-annealing-extension (for set 1: 95°C for 15 sec, 60°C for 40 sec, 72°C for 5 sec and, for set 2: 95°C for 10 sec, 60°C for 10 sec, 72°C for 12 sec). Each sample was run in duplicate. mRNA levels were calculated using the LightCycler 480 software. The mRNA levels of each target gene and the housekeeping gene SF3A, were determined for each sample. PCR amplification efficiencies for all target genes and the housekeeping gene were determined using cDNA dilutions. The relative expression ratio was calculated for set 1 using the delta-delta-Ct method and for set 2 applying a mathematical model incorporating the PCR efficiencies and the crossing point deviation of EGF-treated HeLa cells- versus control non treated cells at each time point.

### Microarrays

#### Agilent

RNA (500 ng) was labeled using Agilent's Low Input RNA Labeling Kit, which involves reverse transcribing the mRNA in the presence of T7-oligo-dT primer to produce cDNA and then in vitro transcribing with T7 RNA polymerase in the presence of Cy3-CTP or Cy5-CTP to produce labeled cRNA. The labeled cRNA of the EGF-treated and the control samples from each biological replicate were labeled with alternate dyes and co-hybridized in duplicate with dye reversal to the Agilent Human 4 × 44K 60-mer oligo microarray according to the manufacturer's protocol. The arrays were washed, dried by centrifugation and scanned on an Agilent G2565BA microarray scanner at 100% PMT and 5 μm resolution. Dual channel Cy5 and Cy3 fluorescence data were extracted using Genepix 6.0 (Molecular Devices) software using the irregular spot finding feature.

#### Operon

Human Operon V4 37K arrays were used featuring 70-mer probes. First and second strand cDNA were synthesized from total RNA (500 ng) with the Aminoallyl Message Amp II Kit (Ambion). cDNA was purified and in vitro transcribed for aRNA synthesis. aRNA was purified and coupled to the Cy ester, and further purified, to remove unincorporated dye. Arrays were hybridized with dye swapping as in Agilent arrays, washed and dried following Operon's instructions on a Maui hybridization station and scanned on an Agilent G2565BA microarray scanner under at 100% PMT and 10 μm resolution. Dual channel Cy5 and Cy3 fluorescence data were extracted using Genepix 6.0 (Molecular Devices) software using the irregular spot finding feature.

#### Illumina

Biotinylated cRNA was prepared using the Illumina RNA Amplification Kit (Ambion) according to the manufacturer's instructions starting with from 200 ng total RNA from each sample. cRNA was purified and each sample was hybridized once on 55-mer probe 48 K Illumina Human WG-6 V 2.0 Expression BeadChips following the manufacturer's instructions. After 16 h of hybridization arrays were washed, dried, stained with Cy3-Streptavidin and scanned using Illumina BeadScan software on the Illumina BeadArray scanning system. Single channel Cy3 fluorescence data were extracted using BeadStudio data analysis software with default settings.

### Digital gene expression (DGE) profiling by high throughput tag sequencing

For each sample, 2 μg of total RNA were used following Illumina's protocol for sequencing of DGE tags. Briefly, libraries of cDNA fragments were generated by capturing transcripts on oligo-dT beads, followed by synthesis of first and second strand cDNA in situ. Cleavage with *Dpn*II resulted in recovery of the most 3' portion of the cDNA molecules, still attached to beads. A 5' adaptor containing a cut site for the type II restriction endonuclease *Mme*I was ligated to the cDNA. Cleavage with *Mme*I released fragments of 17-18 bp from the beads. Following 3' adapter ligation, the resulting library was enriched by PCR amplification (15 cycles), and purified by PAGE. Sequencing by synthesis was carried out on the Genome Analyzer I (Illumina), as recommended by the manufacturer, for 36 cycles.

Raw data were processed using the Illumina pipeline version 1.3.0. 3' adapters were recognized and trimmed using a script that penalizes mismatches to a lesser extent at read ends, following the distribution of sequencing errors along Illumina DGE reads [52]. Several datasets of reference sequences (RefSeq, GeneID predictions, GenScan predictions, RNAgenes) were reduced in complexity by in silico identification of DpnII cut sites and retrieval of these sequences plus 36 nt flanks on either side. The final mapping step was performed by applying Eland iteratively in order to include all possible product sizes, allowing up to 2 mismatches. The compiled collection of expression tags with removed adapters was initially aligned against the reduced-complexity set of RefSeq entries and the targets reference sequences were filtered as in the microarray probe mapping to exclude any targets corresponding to different gene symbols or with no associated gene symbol. Reads mapping unambiguously were counted for each unique transcript within the reduced-complexity RefSeq reference set. Raw transcript counts were first filtered by removal of RefSeq probes with values smaller than 'mean minus standard error' in at least 90% of the samples, where 'mean = average counts of RefSeq probes corresponding to the same gene within one sample' and 'standard error = standard error of counts of RefSeq probes corresponding to the same gene within one sample'. Subsequently, counts were normalized by making sample-wise total numbers of reads equal to the median total number of reads for all samples. Finally, normalized counts of RefSeq probes corresponding to the same gene (defined by gene symbol) were summed up.

### Cross-mapping between platforms

For the purpose of the comparison and to have consistent up to date annotation we remapped all probes in the different microarray platforms to assign them to gene symbols. For each of the platforms (Agilent, Illumina and Operon) sequences for each probe were mapped to the human reference genome and RefSeq reference transcriptome (hg18 accessed through UCSC). Mapping was done using BLAST, BWA and BOWTIE independently. Only unambiguously mapping probes were selected. All ambiguous probes were discarded. Up to 2 mismatches were allowed to consider differences in probe sequence relative to the reference. These can originate from the disparity of sources of sequence information and genomic annotation used by the different microarray manufacturers and can include natural sequence variation as well as sequencing errors in databases, or artifacts generated during probe design. When mapping to the reference genome, annotation information (GTF from UCSC) was used from the same genome version to create a probe-transcript link ID. We selected probes that could be unambiguously mapped at least once to either the genome (where there was an annotated transcript) or to the reference transcriptome, with the main requirement being that there is an association to an official gene symbol. Transcripts corresponding to genes without official gene symbols were ignored.

In the case where a gene was represented by multiple array-specific probes we took the median log2ratio value of the corresponding probes. For the Illumina GA-I sequencing data, counts of probes representing the same gene were summed up before calculating log2ratio values. We took the intersection of genes in all platforms and merged the corresponding log2ratio data.

Next, we took intersections for all combinations of three platforms, then for all combinations of two platforms and, finally, the probes with no overlap between platforms were also scored. Each time, the corresponding data was appended to the existing data matrix. Hence we end up with a matrix containing data for 20,322 RefSeq genes with known HUGO symbols, the union of genes in all platforms under consideration.

### Statistical Analysis

Log2ratio values were computed for all pairs of control and EGF stimulated samples. This was also done for the one-channel microarray platforms since samples are to be considered as paired due to the study design. Further, this procedure makes one- and two-channel data directly comparable.

Analysis for differential expression on a gene-by-gene basis was done by SAM [53] and limma [54], including correction for multiple testing using the False Discovery Rate (FDR) method.

For cross-platform comparisons Gene Set Enrichment Analysis (GSEA) [37] was applied where the gene set of interest was defined as the list of differentially expressed genes as derived from one platform, and its enrichment among differentially expressed genes within the remaining platforms was tested. In order to further assess comparability between platforms we computed CAT ('concordance at the top') plots as described [40].

We also aimed at defining a consensus list of regulated genes using information from all platforms simultaneously. Since expression measures are not directly comparable between different platforms we used the RankProd approach [39] that is based on differential gene expression ranks. Only genes present in all the platforms under consideration can be included in this analysis. Therefore we applied the RankProd analysis for all combinations of platforms as given by the complete merge data matrix described above. P-value adjustment according to [55] (FDR) was then applied to the union of all genes.

In order to explore the changes in gene expression due to EGF stimulation from a more global point of view, we analyzed 218 KEGG pathways [56] with the GlobalAncova approach [57]. Only genes present in all platforms were used for this analysis. The 196 pathways are all available human pathways that contain at least one of those genes. Since GlobalAncova is quite sensitive, we applied a rather conservative method for multiple testing correction [58]. We further explored the pathways with adjusted p-values < 0.01 with respect to interconnections between them. We propose a network of pathways where an edge corresponds to an overlap of regulated genes between the two respective pathways.

### Network and pathway analysis

Ingenuity pathway analysis 3.1 software (IPA; Ingenuity Systems) was used for evaluating the functional significance of EGF-induced gene profiles. Specified lists of genes identified by RankProd as being affected by EGF were used for network generation and pathway analyses implemented in IPA tools. HUGO official gene symbols for the selected gene lists were uploaded into the IPA suite, which were then mapped to the Ingenuity Pathway Knowledge Base. The so-called focus genes were then used for generating biological networks. A score was generated for each network according to the fit of the original set of significant genes. This score reflects the negative logarithm of the *p-*value, which indicates the likelihood for the focus genes in a network of being found together due to random chance. Using a 99% confidence level, scores of ≥2 were considered significant. Significances for biological functions were then assigned to each network by determining a *p-*value for the enrichment of the genes in the network for such functions compared with the whole Ingenuity Pathway Knowledge Base as a reference set.

## List of abbreviations

EGF: Epidermal Growth Factor; DGE: Digital gene expression profiling; RT-qPCR: Real-time quantitative polymerase chain reaction; GSEA: Gene set enrichment analysis; KEGG: Kyoto Encyclopedia of Genes and Genomes; SAM: Significance analysis of microarrays; SAGE: Serial Analysis of Gene Expression; IPA: Ingenuity Pathway Analysis; *GTF*: Gene Transfer Format; CAT: Concordance at the top; HUGO: Human Genome Organization.

## Competing interests

The authors declare that they have no competing interests.

## Authors' contributions

FL and LS conceived and designed experiments; FL and RP performed cell culture experiments and obtained biological samples; FL, AF and EG performed microarray experiments; AV and EC performed DGE experiments; FL, MH, XP, JL, MI, JCD, MN, RK and LS analyzed data; MB, JAR and HH contributed reagents, materials and/or analytical/technical expertise, SI and AMM assisted with technical expertise; FL and RP performed RT-qPCR experiments; FL and LS wrote the paper. All authors read and approved the final manuscript.

## Supplementary Material

Additional file 1 **Figure S1. Activation of signaling pathways in HeLa cells after EGF stimulation**. Serum-starved HeLa cells were stimulated with EGF at the indicated times in the presence or absence of kinase inhibitors. Total cell extracts were prepared as indicated in Materials and Methods and samples were subjected to SDS-PAGE and immunoblotting using the indicated antibodies (A, C, D). (B) Total RNA was prepared as indicated in Material and Methods and samples were subjected to reverse transcription and RT-qPCR using specific primers for the indicated genes. Experiments were carried out in triplicate and in all cases deviation was lower than 10%. (D) Immunoblots showing ERK and p90rsk phosphorylation on the three sets used for this study. Total ERK was used as a loading control. Click here for file

Additional file 2 **Table S1. Gene lists of SAM test overlap by Venn Diagram of 3 microarray platforms and DGE (provided as word file**). Click here for file

Additional file 3 **Table S2. Table of cross platform GSEA enrichment scores and significance values (provided as word file**). Click here for file

Additional file 4 **Table S3. Table of 20192 genes analyzed by RankProd analysis of microarray data (provided as excel file**). Click here for file

Additional file 5 **Table S4. Table of reads generated by the DGE pipeline for each of the runs**. Summary table of read mapping statistics generated by the DGE pipeline for each of the runs. Click here for file

Additional file 6 **Table S5. Table of 20322 RefSeq genes analyzed by RankProd analysis of microarrays and DGE (provided as excel file**). Click here for file

Additional file 7 **Table S6. Table of 1164 genes found significant by RankProd analysis of microarrays and tag ultrasequencing (provided as excel file**). Click here for file

Additional file 8 **Figure S2. Time course RT-qPCR analysis of potential EGF-regulated mRNAs**. Total RNA samples from serum-starved HeLa cells stimulated with EGF at the indicated times (15 min to 24 h) were subjected to quantitative real-time PCR (see Methods for details). Data represent mean fold induction of at least two independent experiments. SFA3 was used as the reference. (A) The upper panel shows the graphical representation. (B) RT-qPCR Fold Changes and corresponding Fold Changes derived from the three microarray platforms and by ultrasequencing. Click here for file

Additional file 9 **Figure S3. Correlation plot between DGE and microarray log2ratio values**. Comparison of estimated log2ratios from DGE (*Y*-axis) and the average of all three microarray platforms (*X*-axis). We consider only genes that were interrogated using all platforms and genes with a mean number of counts across lanes greater than 0. Genes with counts greater than 32 reads in all samples (colored red or green) or less than 32 reads (black) in at least one sample are shown. (Red dots) Genes called differentially expressed based on DGE data at a 10% FDR by RankProd. (Green dots) Genes not called differentially expressed but above 32 counts. (Inset box) Correlation between technologies is higher when considering only genes above the 32 count detection level than when all genes are included. Click here for file

Additional file 10 **Figure S4. Heat maps of genes found regulated at 6 h after EGF treatment of HeLa cells in our study and known to be related to EGF signaling**. Some genes detected in a subset of all platforms are also included for the sake of completion. (A) Modulators of EGF signaling; (B) non-EGF agonists of EGFR and cytokines linked to the EGF family locus on chromosome 4q13.3; (C) EGF-interacting and related proteins; (D)genes described as early and delayed early response to EGF including DNA and RNA binding proteins; and (E) components of the ERBB receptor endocytosis and intracellular trafficking complexes. Click here for file

Additional file 11 **Figure S5. Metallothionein gene expression after EGF treatment**. Log2ratio of EGF-treated versus untreated heat maps of metallothionein gene expression after EGF treatment in (A) HeLa cells at 6 h as determined in this study using Agilent, Operon, and Illumina microarrays, and DGE sequencing; (B) RT-qPCR for 6 metallothionein family members, (C) metallothioneins in HeLa cells in the time course study by Amit et al using the Affymetrix platform, without replication (relative log2ratios obtained by log2intensity subtraction of the 0 time point value from each time point). Click here for file

Additional file 12 **Figure S6. Pathway analysis based on the Ingenuity Pathway Knowledge base**. The three best ranked networks derived from EGF-regulated genes as determined by the RankProd test were (A) Cell Death, Embryonic Development, Renal and Urological Disease (B) Amino Acid Metabolism, Post-Translational Modification, Small Molecule Biochemistry and (C) Cell Cycle, Cancer, Cardiovascular System Development and Function. Upregulated genes are indicated by red symbols and down-regulated genes by green symbols. The shape of the node denotes the main function of the protein encoded by the gene. Smooth lines indicate interaction between the products of the genes; dashed lines indicate an indirect interaction and lines with an arrow indicate an "acts on" relationship. Regulated genes are shown as grey boxes; non-regulated genes associated with the regulation of some of these genes are shown as white. Click here for file

Additional file 13 **Table S7. Significantly regulated genes associated to regulated KEGG cellular functions as determined by GlobalAncova (supplied as word**). Click here for file

Additional file 14 **Table S8. List of primers used in this study**. Click here for file
