# Supplementary material for: Multiple platform assessment of the EGF dependent transcriptome by microarray and deep tag sequencing analysis
Source: BMC Genomics. 2011 Jun 23;12:326. doi: 10.1186/1471-2164-12-326 (PMC3141672; doi:10.1186/1471-2164-12-326)
Supplement: Additional file 1 — Figure S1. Activation of signaling pathways in HeLa cells after EGF stimulation. Serum-starved HeLa cells were stimulated with EGF at the indicated times in the presence or absence of kinase inhibitors. Total cell extracts were prepared as indicated in Materials and Methods and samples were subjected to SDS-PAGE and immunoblotting using the indicated antibodies (A, C, D). (B) Total RNA was prepared as indicated in Material and Methods and samples were subjected to reverse transcription and RT-qPCR using specific primers for the indicated genes. Experiments were carried out in triplicate and in all cases deviation was lower than 10%. (D) Immunoblots showing ERK and p90rsk phosphorylation on the three sets used for this study. Total ERK was used as a loading control. [file 1471-2164-12-326-S1.PPT]

## Slide 1
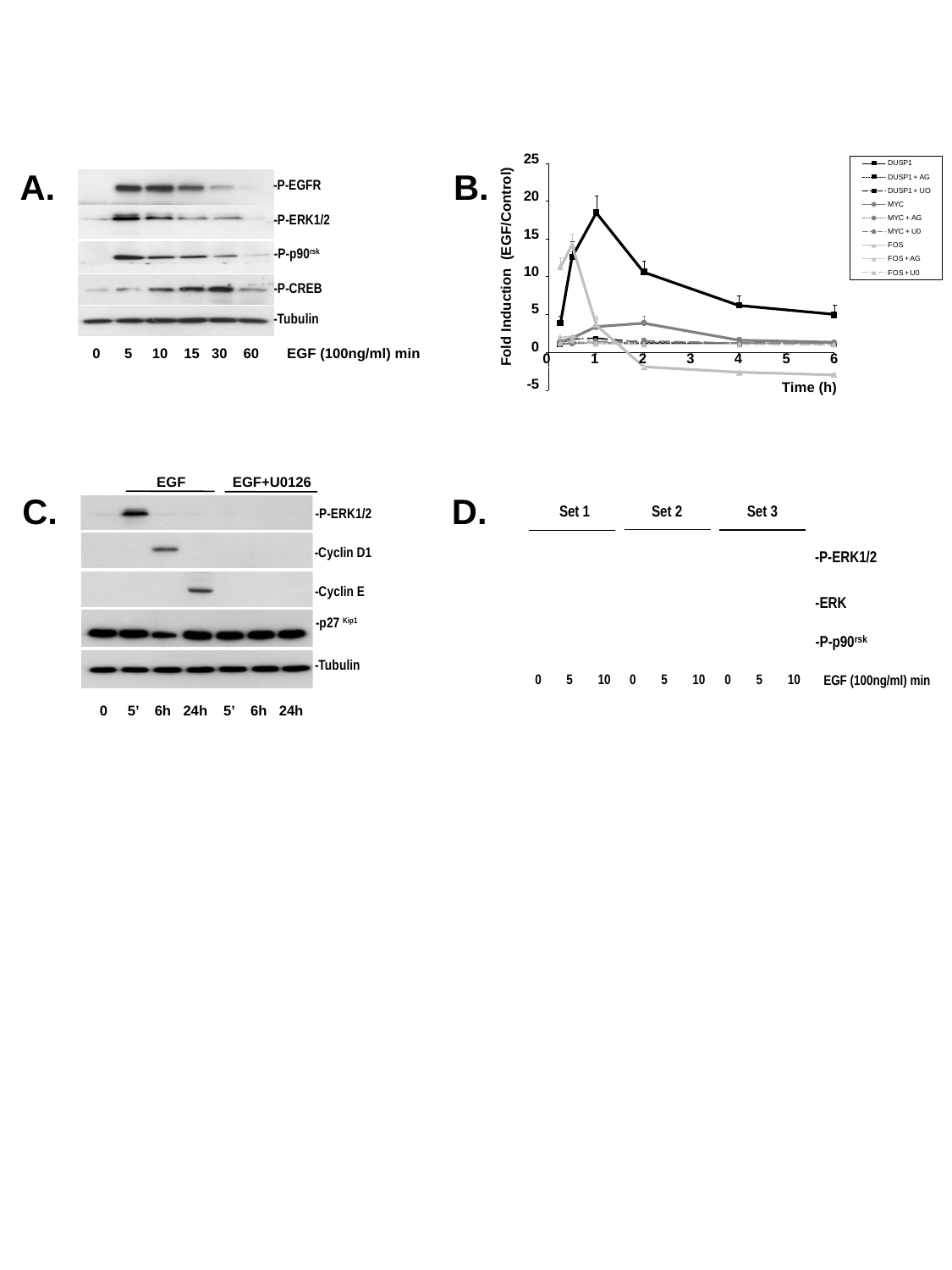

25
A.
B.
-P-EGFR
-P-ERK1/2
-P-p90rsk
-P-CREB
-Tubulin
0 5 10 15 30 60 EGF (100ng/ml) min
20
15
Fold Induction (EGF/Control)
10
5
0
0 1 2 3 4 5 6
-5
Time (h)
EGF
EGF+U0126
C.
D.
-P-ERK1/2
-Cyclin D1
-Cyclin E
-p27 Kip1
-Tubulin
0 5’ 6h 24h 5’ 6h 24h
