# Supplementary material for: Multiple platform assessment of the EGF dependent transcriptome by microarray and deep tag sequencing analysis
Source: BMC Genomics. 2011 Jun 23;12:326. doi: 10.1186/1471-2164-12-326 (PMC3141672; doi:10.1186/1471-2164-12-326)
Supplement: Additional file 2 — Table S1. Gene lists of SAM test overlap by Venn Diagram of 3 microarray platforms and DGE (provided as word file). [file 1471-2164-12-326-S2.DOC]

| Agilent_SAM_UP_1124  AREG EMP1 MT1X MT1L MT1E EREG ITGA2 MT2A MT1B MT1G PTGS2 LYPD3 PHLDA1 IL8 IGFBP1 F3 RGS2 ISG20 GRAMD1B FST MT1H EPHA2 SERPINB1 JUN IER3 IL11 ANGPTL4 FAM83A ADM IGFBP4 UPP1 B3GNT5 CREM GLIPR1 TNS4 CXCL1 SLCO4A1 IL23A ANXA10 WARS EPGN GPRC5A FEZ2 GLRX ERCC1 KCNK1 JUNB PKIA LRRC8C GPR87 AGPAT9 FLNC PLAUR SPHK1 CXCL2 C5orf62 CGA CNIH CAPN2 BIRC2 MUC13 TPM4 SERPINE2 STC2 BMP6 IL4R ANXA2P1 SMTN ITGA6 FOSL1 PPAP2B PMEPA1 GJB3 VEGFC ANKRD13C FAM107B INPP4B SAT1 MCL1 HRH1 WDR69 STK17A EIF2C2 GPR161 EZR OLR1 DNMBP SHANK2 SNRNP35 BIRC3 ANXA2P3 KLF6 RABGEF1 ZMIZ1 LOC148709 SLC20A1 LAMA4 TCF7 PEA15 PXN LOC648740 MPRIP STAMBPL1 MAP2K3 CLCF1 SLC19A2 LOC100130331 C16orf52 ID1 MBOAT2 S100P PITPNC1 HSPA5 DUSP5 AXL ARNT2 SERPINB8 DRAP1 ANKRD57 CSNK1E TIPARP KCNN4 FHL2 ARHGEF2 PLIN2 PDLIM5 NUP50 YOD1 ZFP36 TNFRSF1A OSMR MALL OBFC2A NHEDC2 ASAM FGFBP1 MGLL SOCS2 PFKP PLIN3 SERPINB5 MTAP ST3GAL1 CCND1 DUSP6 NRIP1 JAG1 SFN BCAR3 RND3 KIAA1949 RIPK4 CXCL3 MORC3 GRAMD3 ENC1 RAB31 OAS1 WDR1 CLDN1 ANKRD13A SPOCD1 PHLDA2 SMOX EMR2 TNFRSF12A ARHGEF18 MID1 EXT1 MLKL KRT17 FAS LOC440944 NDRG1 STARD4 TUBB2A FAT1 GBP3 ANXA2P2 SPRY4 POTEF GADD45A TRIM8 PPARG MGC102966 RASGRP3 LDLR DDX21 RASSF8 IER2 RTN2 TNFRSF21 TNIP2 ZNF503 S100A16 YRDC PABPC4L SLC25A22 GFPT2 CHKA NCRNA00152 HOXA5 EPAS1 EFNB2 LOC644936 SNX22 ACTBL2 MYH9 FLJ36031 FERMT2 SLCO1B3 ENTPD7 DLGAP4 MT1A AMIGO2 DUSP1 SNAPC1 LOC644165 CCDC107 ARHGAP10 FAM59A SECTM1 KIFC3 BNC2 FLJ40504 HMGA2 TBC1D2 EIF5A2 PCTK2 CAPRIN2 UBR1 VIP TNFRSF10A LONRF3 ITPRIP TNFRSF25 BCL10 DDIT3 RGS20 IL27RA CIB1 PTRF CHST11 IER5 LY6K NPC1 FHOD3 BTBD10 S100A11 EED CSRNP1 IFNGR1 ARHGEF4 INPP1 UBE2H LOC100132831 PHF21B PPP4R1 EPN2 TMBIM1 ACTG1 SOD2 MAP1LC3B ASB6 STX11 RASSF1 MAFF PLEKHB2 CDC37 sep-10 CCDC85C LEPREL1 ETF1 SLC6A10P PANX1 DDX5 LOC400578 FOXN2 SHB PAQR9 CD55 LOC541471 GRPEL1 CNN2 AKAP8 CYP26B1 S100A2 PICALM TAF1A CAP1 CD44 FHOD1 FGF2 ARHGAP12 IGSF8 ABHD6 SGMS2 STX4 RHOF SOCS3 CDKN1C NP NAMPT NEDD4L DUSP4 HBP1 CHMP2B DLX2 DPH3B PPP2CB SLC38A1 TPBG CASP5 SLC25A37 HIF1A PAQR5 AEN CLOCK AMOTL2 PID1 PTGER4 SSH2 TUBB3 KLF5 RASAL2 CXorf38 SLC45A3 KLHL7 ARID3B TMEM41B CORO1C SLC25A32 MAPKBP1 SLC43A3 FER IL6R CDK6 MAMLD1 BCR SLC1A5 GLS NR1D1 KRT18 KLF7 DOK7 ICAM1 EHD4 CD68 GSK3B GKN2 CASP4 ERICH1 TOP1P1 RSPO3 LRRC8B SELK ALG13 ANXA2 THBD CDK2AP2 STAT3 C16orf45 ULBP2 MAT2A RTN4 TNFAIP8 ARHGAP26 AJAP1 BZW1 ODC1 TMEM158 DNAJC3 RAB21 FCHO1 ANTXR2 FERMT1 ZNF114 KIAA1609 CXorf61 LOC641367 FSCN1 VTI1A GNG12 KCNC4 CNRIP1 ACTB TOP1P2 KPNA4 IRF2BP2 CCM2 MICAL2 NAT8L SPINK1 LYAR CD9 CXCR4 CGRRF1 FXR2 CDKN1A FLJ46111 MESDC1 H3F3B SAMD4A TMEM49 GDPD3 ACSL3 SNX9 SLC7A11 TRIM25 TGIF1 C15orf39 PLCD3 GAD2 PRKAA2 KLHL21 SLK TUBB2C HPCA RPS6KA4 TGFB1 BAG3 CSNK1A1L HIST1H3I SERTAD1 CRY1 sep-09 SEMA3B DNAJB9 CRTC2 FLNA CCDC86 ADORA2B C14orf129 TINAGL1 CDKN2B CLEC2B NRP2 PLAA SCML1 SH2D3A TOR1B PPP1R13L CCNYL1 PPTC7 ARC PTS C9orf167 SOS2 ITGA3 CSGALNACT2 HIST2H3A BCL9L KRT15 VKORC1L1 DRAM1 KRT83 RPF2 MAD2L2 PLEKHG4 DUSP5P CUGBP1 FAM49B ARL5B ARHGEF19 SERINC2 ISG20L2 PPEF1 RRM2 GRK5 UBE2D3 FZD5 DNAJB1 GPR172A ITPR3 PRNP MAP3K5 BAIAP2L1 GSPT2 PMAIP1 DDAH1 RHOC PTPN1 MOSPD1 PPP1R15A GPR126 HIST1H3C DKK1 PHLDB3 C10orf47 GKN1 RYBP CPM CNN3 SLC25A24 NIP7 LRIG1 HIST1H3G LANCL3 GALNT2 ZNF335 TFB2M KBTBD2 PDGFA SH3BGRL3 CEP135 RRP9 H3F3C PTPRH MTRF1L EXTL2 ABL2 ADRB2 COTL1 NDUFV2 PLEKHG5 FICD FAM135A TAGLN PLCL2 ZYX RNF126 ASAP2 FOXA1 BTG3 LRRC8E ONECUT2 PTPN12 GDE1 HSD11B2 DDX47 GALNTL4 NET1 GAN LOC643837 ORAI1 ARPC5L UBE2S FAM63B IGHMBP2 REEP3 VASN RRP12 PRICKLE1 C20orf20 ARHGAP5 TPRG1L DIAPH1 AHR ELFN2 SBNO1 RUNX1 IFNE ZDBF2 SGCA MLX SIRT7 TRMT6 ROR1 ZFAND6 SHANK3 PKP2 KRT8 CIRH1A CDC42EP4 TM4SF1 SPSB1 DIP2C SH2B3 BCL3 NCRNA00162 UTP11L DCBLD2 CD3EAP DPH2 ACTN1 SETD1B C7orf40 MANF UGDH PAWR UBASH3B LAT2 AHNAK TPM3 PPM1J DOPEY2 MT1M PLEKHM1 CYP24A1 TLR6 ATP6V0A1 RIC8A ELOVL1 GEM SH2D4A WDFY2 SLC30A1 SPATS2L ECM1 CMTM7 LOC654433 CLIP2 EIF4E KRR1 GNL1 FOSB ABCC1 C4orf32 FAM3C SLC9A3R1 AAMP JMJD6 MYO10 LRRC8D SLC22A4 ATAD3B C1orf31 PHC2 DHX37 TBC1D15 C9orf89 GABARAPL1 HIVEP1 ATG16L1 PMP22 KANK1 VMA21 EPHB2 SIPA1L2 CDKN2AIP MED10 WWTR1 SAMD8 FJX1 SLC2A1 ISL2 LIF TMEM102 DDIT4 RGS17 BHLHE40 ASPH BMP2K ZNF707 SVIL OTUD1 UTP20 NUDT4 CPEB4 LOC554202 NOLC1 KLF3 PRKAB1 PDZD8 PCDH1 BTD COL7A1 RIPK1 C10orf137 SPAG9 SYNJ2 TNIP1 PLCXD1 CNNM4 STK10 MYBL1 FGFRL1 DVL1 VCL INSIG2 CXADR UCK2 EIF5A SLC30A7 SEC61G FGD6 HSPA14 MMP15 SQRDL SLC4A7 SIAH2 HYAL3 SMAGP VAPA DCUN1D5 C9orf140 DRD2 C1orf128 RASGEF1A FBXO45 TCP10L2 SEC24A C1orf116 AKIRIN2 TSPAN9 SMG5 HIST2H3D CA13 YKT6 BCL7B NSUN2 PARD3 C1orf77 ACTN3 C2CD2L USP38 SLC16A6 KRT79 NOP2 RDH13 TFE3 PLEKHG2 C3orf45 GCAT ARL4D FRS2 RCOR1 RBM9 HISPPD1 MT1F PALLD HOXA11 FAM91A1 KCTD10 KCNG3 TRMT1 SH3TC1 JHDM1D KLF9 B4GALT5 RG9MTD1 SHC1 ACTC1 FMNL2 LAMC1 GJB5 VPS37B XPO5 SGTB TRIB1 RRAS2 GPR56 PSEN1 GRPEL2 DPH3 PSMC4 CDR2L LRRC1 CEP170 PITPNB TNFRSF10B ZNF622 SH3RF3 SNX8 KLHL15 RNF217 USP3 MFSD10 PDP1 RASD1 ACOT9 SDCBP DUSP2 PLEKHH3 C9orf53 C1orf63 TNFRSF10D SHQ1 CSGALNACT1 PDK4 PHF10 NAB2 KLRA1 HBEGF E2F4 SDCBP2 C10orf110 WDR35 FAM108C1 SPRY2 ST6GALNAC6 PSD4 MBP PPAT PPFIBP1 S100A10 VEZT RALA UAP1 EXOSC3 ANXA1 ELL CD109 NMBR NAB1 SMCHD1 KRT16 FAM89B DCP1A TOMM40 EHBP1L1 ABHD2 SPTLC2 EXOC8 C1orf124 HPCAL1 LCE1A UBE2F FLVCR1 PELO TPST2 MAP1LC3B2 C9orf5 CACYBP C17orf91 C17orf67 CAB39 RAB7A DNTTIP2 NCEH1 ETV5 SH3RF1 PIGH NKX3-1 FOXF1 TBC1D24 KLF4 SDC1 ELK3 PCGF1 ERCC3 SLC35F2 MAPK6 CSTF2 LOC284441 NHSL1 MSN ANKRD27 MGC70870 PTTG1IP NSFL1C MYBBP1A S1PR1 REXO4 PWWP2B HIST1H3B EPPK1 TEX14 MYO9B TUBA4B GATA2 DR1 SCNN1A TUBB6 MREG ZNF511 RNF182 UBIAD1 KIAA0020 YIPF6 CSNK1A1 SPINK6 PTPRE ZNF259 TBX2 PSMD2 CTRL ANKRD33B RIOK3 ANKRD22 LOC442421 LOC442308 PRSS22 PRDM10 POLR1E SRM TMEM191A ACTA2 FAM83B IPPK PPARGC1B GNG4 FAM60A TMEM39A NEB UTP15 SPRY1 C1orf103 ZNF710 TCOF1 PDCD6 IBTK EHD1 FOXO3 SLC39A2 KCTD9 MTPN PYROXD1 FES FADS3 ITPKC KEL JOSD1 RELA NT5DC3 DCLRE1C ITGA1 CDC34 CMIP ERO1L HAS2 ACOT7 C5orf43 LYN ARL16 IP6K1 FRMD6 GTF3C5 ANKRD13B HSD17B2 ISCA1L HAUS6 TGFA STMN3 TCF7L2 PDZD2 STIP1 TIMP3 CSK ZDHHC9 KLHL18 FAM122B C10orf2 KIAA0284 RAB20 ARHGDIA PBX4 HCRTR1 C2CD2 GABPB1 IFNAR1 NAT13 C3orf52 SS18L2 DOCK5 CSNK1D DPYSL4 SELS SOAT1 ATXN1 NDEL1 WDR3 IL18R1 NEDD4 DUSP14 SERTAD2 TAF13 STX6 PLEKHO2 SARS TPK1 ITGA5 MDM2 ADRM1 PINX1 TRIM16L C8orf76 RNF24 PRKX LRIG3 MYL12B SLC35C1 ARHGAP22 ZNF394 SIRT6 CSNK1A1P IMP4 SBDS LOC100302652 ARID3A PRKY TMEM79 IGF1R RNF44 NXF1 PAPD5 COX6B2 RAB11FIP1 PTPN3 SLC41A2 CDKN2D QSOX1 RICTOR PNPLA8 TRIM27 FAM110C PTGES RIN1 KTI12 EIF4A3 RAB27B MYD88 LOC100268168 TROVE2 PPP2R2A KCTD5 TNFSF9 C1orf55 SIX1 ABCG2 ATP6V1D ELMOD1 TNPO3 DCAF13 CLCN5 NPAT POP7 ARSJ MYC GOLGA7 CDGAP RABEPK PTRH2 RBM18 HNRNPAB ETV4 TFPI HERC4 NQO2 WNT7B RELL1 C11orf82 TUBA3C CTTN SNRPB SP110 MARS2 CLIC1 SNAI1 PTBP1 SPRED2 FA2H LRRC8A LOC493754 CBX4 C16orf72 FRMD8 CBFB SP1 LPCAT1 SMURF2 PPP3CA GOLT1B ZNF670 EFHD2 BICD1 CDH5 NT5E AATF TMEM22 INF2 DUSP7 TRIO C1GALT1C1 TIMP1 LOC723972 FAM176A TRIM11 FZD10 AKIRIN1 DDX10 C16orf57 GPR137B C18orf19 CDYL2 USP53 HOXA3 ANP32C RABIF RPIA SNAPC2 BAK1 FNBP1 DCTPP1 EIF2C3 VHL sep-11 RGS16 FMNL1 CHST15 PUS7L GAP43 ANP32D ENTPD6 KIAA0406 SAP30BP GNAI2 EPHX4 EIF1AD ERI1 CISD2 BCL7A PSD3 GNPNAT1 EBNA1BP2 CENPO HOXC10 CDC14C ADSSL1 PRMT1 PRPF38B COQ10B CBL MAP4K4 ACRC RPGR B3GALNT2 ZCCHC17 KDM2A CEP170L ABTB1 ARHGEF15 TUBB4 RAB25 |
| --- |
| Agilent_SAM_DW_1275  LOC729678 GTF2I PEX13 IQCE ACTR1B PCK2 DENND5B ZNF428 SGMS1 PMPCB ORC3L RNF146 VGLL3 DIRAS1 DDX6 ANXA4 PBX1 ACADS RNASEH2B C16orf88 PHF11 PEX6 LOC100288778 SLFN12 FBXO22 SYT12 DLG5 CDCA7 EFR3A KIAA1586 COL4A3BP STOX1 CRIPAK PCMTD1 CYP20A1 LPIN1 FECH USP48 ACBD6 DUT LSM8 CKAP2 CD19 HNRNPL SETBP1 C3orf34 LOC653113 ADO ARVCF FIGF PPL C1orf115 STRADB TSKS LYRM7 SLC2A8 MLYCD ZNF451 SNW1 DAND5 ASXL1 SLC22A11 POT1 PLK2 STX17 RNF219 SLC2A4 ENDOG NDUFS2 ZNHIT3 VPS45 KIAA0319L MMP11 E2F2 OGFRL1 PEX12 ZNF37A EIF4E2 ZBTB47 DIS3L2 CASKIN2 DCAF8 DRD3 CSRNP2 CAPS ZBTB33 IGF2BP1 ZC3H8 SAT2 C4orf41 RG9MTD3 LRTOMT POLD1 UNC119B TMCO3 CCDC74A IFT81 CCDC28A SPC25 ANO8 IDH1 USP20 USP30 ACOT13 ABLIM1 CRYZL1 ZNF354B C22orf46 COPG2 FAM111A ATF3 NUPR1 TSNAX CEP70 KLHDC3 TTC30A ZNF398 WDR81 STX12 STAT6 SMUG1 AFF1 ADAT1 C7orf27 TK2 ACSS2 KIAA0195 GSTT2 ACSF3 BZRAP1 DYNC1LI2 CDC7 RUFY1 ZNF271 POMT2 NUDT9P1 WWC2 OIP5 RNF150 KLHL12 NT5M CRLS1 MLLT3 FAM102B CCDC85A C17orf95 ARMCX3 CCDC113 NDUFA5 HSD17B4 NDUFB5 SEC22C KLHL8 ID3 PIGW TBC1D7 IMPDH2 A2LD1 RHOU DHRS12 ORAI3 NDRG2 ACADM CBR4 WDR7 ZNF768 KIAA1370 C12orf26 TYMS ARL6IP1 TNNC1 C14orf101 GYG1 HIBADH ZNF397 CD70 PPCDC PM20D2 BTG2 LOC613038 HEXIM2 TGFBR3 UNC93B1 SREBF1 TADA2A RBM16 CYP2U1 ZNF654 BEX2 CDC25C KIAA0892 HS6ST1 MRPS14 OPLAH SLC25A23 CENPH DERA AZI1 FDXR MTMR9L RAB28 HERC2 DENND3 SPAG7 NFYB IPO13 DTWD1 RAD17 VPS39 BBS10 POLA1 VPS54 RPUSD3 TP53TG1 FAM35B RAD54L PRPF39 MSH6 PBK KBTBD11 FAM36A NDNL2 MARS USP35 NAT14 C2orf47 RFXAP MANSC1 DIO2 ISOC1 PPP2R3B TNFSF12 THRAP3 KRTAP4-7 C17orf58 ASB13 C15orf52 SNX25 TRMT5 NUP85 RAB26 PLCG2 EIF2AK4 SMC1A ZNF672 ZNF777 PARD6A FUNDC1 NMI NT5DC1 LETMD1 PQLC3 R3HDM2 UFSP2 UQCRC2 C22orf36 SHMT1 LRP12 KCNQ1 PDXK OSBPL1A TIMELESS ZNF383 IGBP1 EPC2 HIST1H4F WNT10A ELP4 GLMN DCI LOC389705 RNF113A INVS FGF12 SMAD1 C8orf40 FPGT FARP1 CDAN1 PEX14 EFNA4 DENND2D C20orf108 SNX7 RASL11A UCRC GCN1L1 PKD2 TAF1D ZNF396 C16orf93 MBLAC1 C15orf40 SUMF1 WDR90 ARID2 SRR CCR10 APBB3 GAS2L1 RRP8 HOOK2 HMGB1L1 SAP30 LOC729991 RASSF2 SLC31A2 HDAC4 FAM165B RNF141 HOXA11AS HNRPLL GGA3 NCRNA00087 RNF128 PROCA1 CCDC125 CMAS DUSP3 C12orf47 NUP107 AP3M2 EPM2AIP1 HIST2H4B YEATS4 MBOAT1 KLHL22 ATRIP PPP3CB ZNF148 TMTC3 KIAA0586 OBSL1 LCA5 ZNF37B SAC3D1 WDR92 SLC35B3 SLFN11 CEPT1 CDNF RIMBP3C TSNAXIP1 LOC653501 TXNDC15 PGAP3 PPP1R8 ACAT1 CFL2 C2orf15 L3MBTL TMEM80 REL MRPS18B ZNF7 MRPL38 WBP1 RBL2 ACP6 ARMC1 FAM105A SLC43A2 CHMP4A ZMYM1 TMEM8B DLG3 MDC1 TTLL1 KIF11 PIGZ RNASEH2A MTIF3 KANK2 ZNF362 MRPL34 LIPT1 P2RY2 GPRASP2 PNRC2 AKR1C1 SCCPDH CTDSP2 LOC100128164 GAS8 THAP8 SRI ZNF248 ZNF277 UBA3 TCEAL2 HEXDC ZNF354A ZNF18 HSDL1 AIMP2 LRFN3 CDK9 C1orf83 FAM45B FZD2 SNAPC5 MMACHC MIA3 KRBA1 COX4NB BET1 SH3YL1 MPG TERF1 DNAJB5 CRADD GOLSYN SH3BGRL2 PPM1B C5orf30 FBXL15 TUBGCP5 MED22 C5orf53 TATDN3 MVD PRSS16 C14orf133 NMRAL1 C9orf103 ZUFSP CYP1B1 CALML6 CYTSB FBXO32 C14orf169 LTA4H DEPDC1 SCRN3 POLR3C PPP1R3E PAIP2B MRFAP1L1 ZDHHC4 C21orf33 CD24 TP53INP2 BSDC1 NR2F1 AVEN MRM1 PAQR6 C20orf94 ZBTB25 BTBD3 HAUS4 COL1A1 CHDH SLC24A1 ASAP3 RAD51AP1 HCFC2 CCS NFU1 IVNS1ABP ALDH3A2 LOC389493 TMTC4 DFNB59 SFRS12 TBC1D8B RANBP10 BBS4 C6orf48 TWIST1 DNAJB14 SLC25A35 ZMAT2 CAMK2G RNF135 ZFX TCEAL1 KATNAL2 PXMP3 MTA3 KAT2A FAM173B ARHGAP28 PNPLA4 CPPED1 ZNF862 SPATA7 RALGAPB STAU2 OSBPL5 FAM45A PYCRL SSH3 CAMLG ZNF512B CETN3 TCEAL4 CDC23 CREG2 COQ7 ENOX2 MICALL2 AMPD3 SNAPC3 KIAA0528 PDGFRL SPR POLD3 TMEM149 CCDC90B SMC2 CCDC110 C6orf125 EID2B MAP3K7IP1 MRPS26 IAH1 LEO1 PKP4 ZNF226 WNT5A C2orf43 MEGF6 CAPS2 HOXA13 LDOC1L C10orf75 PDF BCLAF1 PBXIP1 ETAA1 ERAL1 EID2 PHTF1 NUAK1 ZNF280D ACSF2 C1orf66 PER1 CCDC41 CDK5RAP3 THAP11 GALNT7 SLC38A9 KNTC1 IQCD RAB7L1 AKR1C2 DFNB31 AHSA2 KIAA1737 RPAP3 ZNF285A EPB41L4A LOC100128822 ALDH9A1 CARD8 SNX24 PGBD3 PPIC ANAPC7 RPAP1 ZSCAN21 HLCS PRKCDBP RABL2A CCDC126 DOLK SLC35A1 ZNF571 C11orf75 BRD8 REPIN1 LOC728661 NRXN3 ADD3 CTBP1 C17orf68 DYNC2LI1 MUTED LSM10 NUCKS1 IRAK1BP1 MND1 ITPR1 SGPP1 C4orf27 SNHG7 GPSM2 COX7A2L MBIP TSFM TMEM128 CDK5RAP2 ROBLD3 VPS72 IFT140 INPPL1 LDHD MST1 FAM71E1 TTC13 TLR3 C14orf80 NEURL1B PIGC KAZALD1 A2M HIRIP3 NEDD9 PDIK1L TIMM9 EPS8L2 PHLDB1 RABL5 OCEL1 RPAIN PIPSL PCM1 RAD21 LSS LOC645676 CITED2 EID3 PLEKHA5 C19orf48 BIVM DNMT3B BLMH CCNG1 C16orf48 ZFAT DUS4L LIPT2 TDRD3 NTF4 C9orf125 MAGEA11 C4orf52 DOPEY1 COQ2 FAM96A MLXIPL ICT1 GMPR2 RNASEL C6orf70 CD99L2 TBC1D9B ABCC5 SFI1 NKX3-2 FLJ37453 SASS6 CCT6B PPRC1 SLC25A12 FAM149A MYO5C TACO1 C11orf17 ARMC4 TRUB2 HERC2P2 RBM41 VLDLR FAM35B2 PHF15 CCDC53 PEX1 SPRYD4 GALM ABHD4 ELAC1 LRRC45 DEPDC5 KLHL3 CCDC102A ZNF239 STOX2 NUDT9 HPS6 C6orf211 WDR34 PDK2 FAM192A MAP3K10 CPSF3 ARMCX5 INSL4 C6orf89 ZNF337 NINL FTSJD2 TSEN15 ARMCX2 CTNNBIP1 FANCA ATP1B1 TMEM17 KIF15 SCARA3 SNX29 ZNF580 BFSP1 CCHCR1 TM2D3 FOXN4 THTPA SH3BP4 C7orf49 CYBASC3 SUSD2 NMNAT3 TRAF3IP1 TMEM115 PET112L DDX50 CHCHD4 ZNF589 PLEKHG3 PLD1 CCDC78 CAV2 PAIP1 ANKRD32 RAB11FIP3 CCDC117 AKR1C3 VARS2 THNSL2 ROR2 RAD50 PHIP FAM195A DPPA2 GLRB NOXA1 KBTBD7 ALDH3B1 NRTN PLAGL2 MMAB FBXO16 C1orf123 PPP2R2B MTR SRBD1 C6orf192 WDHD1 FAHD1 OAZ2 PACSIN3 KIF20A SMARCA2 TTC25 CHEK2 MARCH5 HESX1 MTIF2 KIAA1522 AHRR PSIP1 RUNDC3B XRCC6BP1 LATS2 MRPL9 NUSAP1 CACNB2 CCNA1 TCEAL8 IMPACT KIAA1712 TSPYL4 STX18 DHX30 C9orf9 HELZ LOC401431 LIAS C16orf59 ZNF524 SLC2A11 MCM2 P2RY6 NPY1R ZNF33B PLEKHA1 PIGY ARHGEF17 AMZ2 TP53 MRPS31 SNTB2 PARP2 ARID5B MKS1 DMAP1 CCDC14 TMEM170B LOC100093631 AGGF1 ING3 ANO2 PDCL NUF2 TTC12 CXXC4 PPCS ACTR10 HERC1 PCSK4 RNF20 MSRB2 PLAC2 C15orf27 PLEKHJ1 RBM12 KIAA1430 SH3BGR APITD1 ARL2BP MRPL1 MN1 COMTD1 LMO1 XYLT2 GBF1 MORF4 MORF4L1 ADCK2 MRPS23 GALNT11 USP40 N4BP2L2 WDR75 JDP2 IL17RB PCMTD2 CDKN2C TLCD1 BBS2 ZFP62 RASSF4 MITF NFIL3 HMBS ASPM ANKLE2 MDM1 DAPK2 TMEM218 SESN1 NBR1 CCDC111 SLTM RARB C18orf56 C14orf93 E4F1 NFE2L2 MCM3 C6orf130 RAB17 C19orf21 ANKH DDX23 CYB5D2 WDR24 C2orf74 LARS2 MRPS11 CASP6 KIAA1009 NEK9 BRMS1L AKAP9 ZNF76 PRPF40B STX16 OGDHL C4orf34 C17orf42 XPA PAAF1 DNAJC19 HNRPDL AIMP1 GNAZ SETDB2 ZNF462 BASP1 ADA SYNC ADAP2 METTL12 THYN1 TMEM106B TTC30B ATP6AP1L C17orf90 MLH1 ACOT2 RDX MRPL10 TRADD DHX32 XPC VNN2 PARP4 PLSCR4 LACTB C22orf39 SYNE1 FLJ45244 ZBED5 RDM1 PIGQ GRB14 CAV1 C7orf25 CUL9 MNS1 ACCN2 ARRDC4 DDX28 WNK1 C5orf54 PRMT7 KDELC1 LQK1 WDR44 COBLL1 PTPRA GLOD4 LACE1 ZNF219 RCCD1 COPS4 ITFG3 ZCCHC11 C18orf55 SNX17 GLCCI1 LOC100270746 PSMG2 AASDHPPT COQ5 NKAP SIRT3 ZFP112 EFCAB7 IPP CDT1 MGRN1 TAF6 GPR37 CLTCL1 ASTE1 FBXO4 MED20 MCCC1 HAUS1 TMEM138 CTDSPL EIF4A2 C5orf42 PC CBY1 B3GNT1 BRCA1 COQ9 HPS4 PTEN NICN1 ZNF33A KIAA0776 CCDC56 WBP5 ZNF322A LIG1 JAG2 CHAF1B C22orf29 RBM5 RICH2 PHOSPHO2 XRCC1 MYLK LSM6 ABCB6 PPAP2A ATR FAM35A ZNF658 NSMCE4A ABCD4 SMARCAL1 FANCC SIK2 PARP16 SP5 C21orf125 PSMD3 CITED4 DEM1 NMT1 ABCB7 NR1H3 ABCA5 RAB32 DDX59 TMEM101 PSMD11 DET1 C7orf11 ZHX3 ASZ1 DLGAP5 VIPR2 FOXN3 IFT172 PMS1 SEC31B OMA1 MRPL15 RIMS3 C1RL HSPA2 BAI2 CRB1 TRIM6 C20orf29 PNRC1 TMEM107 C2orf64 LOC340508 CTU2 GKAP1 FXR1 C1orf59 KLHL9 FAM69B SNX2 ARAP3 RMI1 CCDC25 C11orf93 SF4 CYR61 WIZ PCSK1 PIR CPT2 C1orf25 CIT SACM1L FAM175A SPINK5 FANCL NFKBIA GOLGA5 FYCO1 MEGF9 PI4K2B SNHG1 FAM185A C5orf13 TNS3 HDHD2 PRMT6 KDM3B ZNF32 PAN2 KLHL24 MRPL46 PEX11B NPHP4 SNHG8 RWDD2B SPIN2B JUB ATG4C TSC22D3 SNCA METTL7A CCNB2 DSN1 COX15 POLR3GL FAIM PRSS23 C2orf68 ZBED3 HOXB13 RWDD3 C3orf18 POLI IFT74 C10orf57 C6orf120 TTC8 NAT1 TMEM120B RTN4IP1 AARS IDI1 RDH11 KIAA1279 DCPS COQ10A CRTC3 PSMD10 FAM175B OSGEPL1 C6orf26 SPTAN1 CENPF SMARCE1 NEURL4 PRIM1 C16orf75 GAS1 RECK KRCC1 SPSB2 COG2 SNHG12 SALL2 ENO3 TRIM45 MTERFD3 TMPRSS5 SH3D19 C1QTNF6 BRD3 DHRS3 FASTK NUAK2 F8A1 TEX19 DDB2 VSIG10 ARV1 CABC1 HS1BP3 NR4A2 FLJ10357 VPS33B NEAT1 NCRNA00094 AGXT2L1 CEBPD TMEM177 BIRC5 PDCD4 NUDT18 FANCG HARS2 NUDT7 CLUAP1 RDH10 COL9A3 AMBRA1 LARP6 GLT8D1 ERBB2 DIS3L SYNM MOSC1 MGC23284 C6orf203 HMGCL NUDT12 AMACR FBXL16 C6orf154 DPH5 C9orf23 H1FX NEK2 C3orf31 MAP2K6 NVL FSTL3 PSMD7 TMEM37 NSL1 TFRC TBC1D9 PRKD3 D2HGDH TIGD2 RFK GEMIN6 PELI3 ATP6V1E2 HEATR5A DCP1B ZC3H6 TRIM66 FLYWCH2 SAMD11 ZNF323 RHOBTB1 C17orf108 ULK1 BMP4 MPI GPKOW C5orf33 C19orf57 DLX4 PIGV LRRC20 ACOT1 KLHDC2 ATG16L2 ZNF573 CREB3L4 HSPB3 PDLIM3 DNAJC18 ECHDC3 PPAPDC2 TBX1 FAM78A TMEM42 COL16A1 RDH14 GADD45B WDR70 LGSN NR4A3 NR4A1 CRBN SALL4 SLC7A6OS PDE8B ARHGAP18 BDKRB2 OSCP1 LOC148413 C12orf76 NTN4 MARVELD1 SMCR7 CACNG6 CTTNBP2 PCYOX1L DDIT4L PPAPDC3 HRCT1 EFR3B ELF3 C6orf176 CSRP2BP PSKH1 LMO4 BACE1 BOLA1 PLK1S1 RHOBTB2 UNK PIK3C2B NLRX1 KCNIP3 TMEM129 CCDC85B TNFSF10 RHOV LOC154822 TMEM187 SORBS2 FOS |
| Operon_SAM_UP_431  IL8 AREG EREG IL11 PTGS2 PKIA ISG20 EMP1 LYPD3 GLIPR1 ANGPTL4 VIP MT1H F3 PHLDA1 UPP1 RGS2 SERPINE2 GLRX FEZ2 GPR87 KCNC4 EPHA2 SERPINB1 FST PHLDA2 BIRC2 KCNK1 MT2A WARS PITPNC1 IER3 ANXA10 CSNK1E EPGN UBASH3B CGA NHEDC2 ID1 CHRNG FAM107B SERPINB5 MT1B MTAP SDC4 SPHK1 PANX1 TUBB2A ADM STX11 ANXA2P1 LRRC8C ANKRD57 CAPN2 MYADM MBOAT2 STC2 ANKRD13C CNIH TNFRSF1A PLAUR SLC20A1 NT5E IGFBP4 KRT15 KLF6 ANXA2P3 MT1X CREM ANXA2 IER5 SPINK1 SOD2 AXL AGPAT9 PLIN3 VGF FAM83A sep-10 MCL1 GFPT2 HRH1 DUSP1 CLDN1 ANKRD13A STAMBPL1 S100P ARC IER2 BMP6 TUBB3 PPTC7 NAMPT ANXA2P2 ERCC1 KRT17 ARL5B SOCS2 OLR1 RND3 NP PFKP PDLIM5 CLCF1 OSTbeta HOXA5 BCAR3 TPM4 BCL9L TIPARP FAM47A EIF2C2 PICALM BZW1 EZR BZW1L1 PMEPA1 PMAIP1 KLF5 NT5DC3 MPZL2 CXCL3 KRT12 SBNO1 KIFC3 ULBP2 TROVE2 GDE1 SERPINB8 OSMR RGS17 PPP1R15A EGR1 LONRF3 KRT8 SLCO4A1 MLKL SFN ODC1 PNPLA8 TMEM158 PPARG TTC32 GRPEL1 TPBG LY6K RASSF8 LYAR GADD45A FGF2 SLC2A1 FHDC1 TNFRSF10D MALL ASAM RAB31 C17orf91 CD9 FERMT2 DOK7 EPHX4 RHEBL1 PLIN2 PLEC1 TNFAIP8 MT1M UBE2H GPRC5A S100A11 HOXA1 OBFC2A STMN3 WDR1 C16orf52 RASGEF1A DLX2 CIB1 COX6B2 KRT18 ARHGAP12 GLS SLK CSGALNACT2 B3GNT5 DIS3 MAT2A TRIB1 RABGEF1 PPP2CB DRAP1 ATXN1 FGFBP1 GKN1 EIF4E FAT1 TAGLN CNN3 GBP3 ITGA5 FCHO1 EXTL2 SLC30A1 RIOK3 IFNGR1 CDKN1C NDRG1 MOSPD1 SDC1 DUSP5 TRMT6 UBE2D1 C4orf32 SCML1 HBEGF SLC25A37 YOD1 WDR69 EXT1 TRPC1 TEX14 CCDC85C RPL13AP6 STK4 CCM2 ACTG1 GRAMD3 KLHDC10 UBE2F PDK4 ENC1 YRDC UTP11L SERINC2 ABHD2 DDX21 PIGH STARD4 ACTN1 CHMP2B KCNG4 TMED7-TICAM2 UAP1 C17orf70 ABTB1 CRTC2 TUBBP5 S100A2 CHORDC1 ELL2 PLCL2 PLEKHB2 GNG12 IRGM MIDN FGFRL1 MAP1LC3B CXADR PRNP BCL10 PTS TMEM49 ANXA1 DDIT4 TMEFF1 TGDS AMOTL2 SYCE1 GPR161 TYMP FAS DUSP14 GJB5 SHISA3 SLC25A32 PLAA RAB21 RRAS2 C5orf62 CCND1 FNDC3A CSNK1A1 GPR172A CCDC107 DCLK1 MAP1LC3B2 REEP3 DNAJC5 BTBD10 FHOD1 CHST11 ETF1 CDH2 TAF1A SLC22A4 PID1 SLCO1B3 RRP15 INPP1 RBM34 ITGA6 PRR23C PIGL TFB2M CD55 UCK2 GRAMD1B NAB1 ADARB1 ITPKC DNAJC3 NPC1 ATP6V1C1 JMJD1C INSIG2 MLX KIAA1609 C18orf19 POTEF UBE2D3 HOXA10 LAYN VKORC1L1 SEMA3B TUBB2C MAPK6 CDK2AP2 PHF10 SGMS2 DDIT3 ELOVL6 UGDH RPS6KA3 C5orf43 LRRC59 RAB20 ALG13 SELK KBTBD2 RASSF1 PTRH2 IGSF8 TOR1B NR0B2 FAM129B SLC7A11 PEA15 HBP1 KRR1 ITGA1 WDFY2 TNFAIP3 SLC41A2 BTG3 PCK1 DNAJB9 MGLL CXADRP2 FAM135A CDC37 RBM18 CA13 ZNF469 BASE SERTAD1 FAM89B C19orf22 HPCAL1 USP38 DPH3B PMP22 BIRC3 KLF9 TMBIM1 C7orf16 ALOX5AP AFAP1L1 EIF5A2 C9orf89 HSPA14 FAM3C WWTR1 MFSD8 GKN2 PTPN3 FJX1 CDKN2B ZNF643 FLVCR1 CACYBP BRSK2 RABEPK RPS6KA6 CXCR4 FAM60A ELOVL1 ITGB1 CAPZA1 RRP9 LIF PCDH1 LIG4 RIOK1 psiTPTE22 MAD2L2 LOC643837 STC1 FAM102A GCH1 FSCN1 PELO ZNF267 AKIRIN2 TNFSF12 SPATS2L GPR126 HSPH1 DR1 RNF149 SPATA5L1 CCDC82 TNKS2 TRIM25 CD59 C12orf59 CAB39 HNRNPU SURF6 NUFIP2 SERTAD2 C16orf90 MED10 |
| Operon_SAM_DW_358  THRAP3 LSM8 PHIP FAM195A CCDC25 PSMD11 FBXO4 UQCRC2 SLC9A6 SP140 ANKRD32 STX17 CCNA1 PDE3B TRUB2 IP6K2 IQGAP3 CPSF3 DAG1 HSPA2 NFS1 DMAP1 C1orf123 NIPSNAP1 MAN1B1 KIAA0776 FBRSL1 POLR2A GDAP1 SMPD1 SLC27A3 LYRM7 CSRP2BP ABCA8 C4orf52 MTIF2 GKAP1 HERC2P2 NR4A1 CAPS KLHL8 MXD3 THAP11 AMZ2 LOC283070 ATR ATXN2 SPATA17 DIRAS1 CBY1 MIF4GD C1orf115 CTBP1 CHCHD4 EPRS CCDC110 COMTD1 HMGB3 NUAK2 SGSM2 BCLAF1 C6orf130 HAUS4 C6orf226 NUP93 MZF1 SMC2 DLGAP5 GLT8D1 CYTH3 APBB1 TRADD C7orf49 COQ5 SPATA4 IGBP1 RNF166 HP1BP3 BRD3 FLJ39739 ASH2L RAB11FIP2 CITED4 HSPC157 ARFGEF1 CDT1 ADD3 KIF20A RUFY1 TMEM106B FBXO43 EFCAB7 MAP2K5 CD24 C9orf86 C14orf126 SNX17 TST THAP8 DDB2 TBC1D8B HDHD2 ZNF500 NUDT8 BBC3 WDR44 DDX59 PMS1 DAPK2 C8orf40 SNX2 GYLTL1B RBM5 C21orf33 CDC23 RHOU RDX CCNB2 C1QTNF6 SEPP1 KIAA1586 ACLY ACADS PDXK C17orf85 FAIM UROS CBR4 NOXA1 C4orf34 PAIP1 CEBPD C1orf59 PCMTD2 IMPACT RGL3 XPA PTEN HPS6 AP3M2 C15orf40 NTN4 WIZ PPAPDC3 DRG2 SPAG16 AES FAM35A DSN1 FCHSD2 TGFBR3 TRUB1 ZNF473 WDR78 CHAF1B ADAT1 ABCA5 PDE5A BBS2 ENO3 NR4A2 PSMD10 SMARCE1 CEP78 MRPL46 GOLGA5 ZNF774 BBS10 CCDC113 PAQR8 C18orf55 ACOT1 GPKOW C7orf11 LOC284551 BTBD3 CACNA2D3 STAU2 FANCL IFT81 UCN ECHDC3 KIF11 RMI1 CCDC53 PER3 SCAMP5 PDIK1L CAV1 MCM2 CITED2 E4F1 SLC7A6OS FAM175A TTC30A RDH14 PRSS23 KIAA1009 WDR70 PHOSPHO2 MRPS16 H1FX TAF9B DDB1 FAM45B TP53INP1 PACSIN3 FAM149A PPL TMEM139 ATG16L2 SF4 EFNA4 CPT2 TMEM107 ZNF204 UNC93B1 POMT2 PARD6A PSMD4 DPH5 TACO1 PLSCR4 RARB FAM125B GEMIN6 TMEM129 OMA1 TLCD1 ASTE1 SPTAN1 TSC22D3 LANCL1 RNPEPL1 NEK2 PSKH1 SYNC R3HDM2 DNAJC16 IFT74 CCHCR1 BIVM PYCRL ARV1 C9orf23 ZYG11B ZMYM1 ZNF350 XPC PLAC8 FANCC KDELC2 SPSB3 ZNF451 MAP2K6 CDC2L6 CCDC126 COL16A1 ZNF573 AKR1C3 TMEM42 TIGD2 TARDBP NUP43 MCM5 SNHG12 C1orf25 TMEM175 MARVELD1 HIST3H2A AARS NFKBIA CENPF SLC29A3 STAT2 PPRC1 POLR3GL ALPP ZNF33A ANO8 PRMT7 NMNAT3 DHX32 AGXT2L1 PDCD4 C3orf31 ANKH NSMCE4A ASB13 CYB5D2 GPR37 FLYWCH2 BAI2 HEXDC TFRC JDP2 CDCA7L ZXDB TRIM6 ARMC1 MEGF9 SCARA3 CCDC14 SLC25A12 ZBED3 KLHDC2 LOC148413 COQ9 SP5 GADD45B LOC647946 CTDSPL C17orf42 ADCK2 HEATR5A RHOV PRIM1 C6orf203 ACSS2 ABCC5 PLK1S1 HDAC4 CCDC111 ARHGAP18 TMEM177 MOSC1 HIST1H4C BOLA1 C5orf33 ASZ1 KLHL22 RDH10 C6orf176 MN1 MRPS26 LOC100128811 CYBASC3 HSPB3 TXNIP ASPHD2 DUSP3 CREB3L4 CCDC85B DDIT4L ZNF323 TNFSF10 PRKD3 RFK ELF3 PIK3C2B ZNF837 LMO4 NEAT1 VIPR2 ID3 ORAI3 RAB26 FOS |
| Illumina_SAM_UP_510  EMP1 IL11 ITGA2 LYPD3 MT2A MT1A PHLDA1 F3 ISG20 FAM83A IL8 IER3 EPHA2 PTGS2 ANTXR2 SDC4 LRRC8C GLIPR1 MT1X SERPINB1 IGFBP4 SERPINE2 WARS ETS1 FOSL1 ADM AGPAT9 GPRC5A RGS2 PHLDA2 VIP EREG MUC13 GLRX CLCF1 ANKRD57 NDRG1 KLF6 FST ANXA10 SLCO4A1 ANXA2 PKIA PLAUR EFNB2 TPM4 AXL FAM107B CSNK1E ANGPTL4 ERRFI1 YOD1 ARHGEF2 TAGLN IER5 TUBB3 FLNC ERCC1 OSMR CAPRIN2 BIRC2 PMEPA1 PITPNC1 BMP6 SMTN SERPINB8 DUSP5 ZFP36 CAPN2 VEGFC HOXA5 IGFBP1 ITGA5 TRIB1 MIDN PKP2 EXT1 CDKN1C ANXA2P3 CYP26B1 URG4 S100P SOCS2 PEA15 TNFRSF1A SFN ANXA2P1 OBFC2A PPP1R15A SOD2 STAMBPL1 IER2 SHB PLIN3 KCTD5 MTAP KCNN4 SAT1 SAMD4A ID1 CNIH CSRNP1 MYH9 NP HK2 FERMT1 KIAA1949 RASD1 AMY1B SEMA4B ZNF503 S100A16 MID1 RIPK4 TBC1D2 GADD45A PLIN2 RAB31 IL4R BCAR3 TNFRSF10A FEZ2 ARHGEF18 LY6K MPRIP GFPT2 UBE2H FGFBP1 CIB1 OLR1 ANKRD13A EHD4 EMR2 SLC25A37 TNFRSF10D PFKP TMBIM1 sep-10 MCL1 DRAP1 PLCD3 IFNGR1 EZR SEMA3B INPP1 MBOAT2 KLHL21 WDR69 SERINC2 DUSP1 COTL1 WDR1 RND3 TSPAN9 S100A11 ARHGEF19 ENTPD7 KIFC3 CCDC85C CFLAR MLKL INPP4B NPC1 DDIT4 RAP1B CSGALNACT2 MGLL TRIM8 SERTAD1 DNAJC5 GPR161 C16orf57 TNIP2 TIPARP CDC42EP4 SLC2A1 GALNTL4 GSK3B SLC35F2 CDK2AP2 SLC25A22 PICALM GPR172A LEPREL1 ALG13 PMP22 ULBP2 ZNF668 PDLIM5 FMNL2 NKX2-5 ARID3B S100A2 NEDD4L CNN2 FHOD1 BTBD10 FOXN2 CD9 ARHGEF4 TNFRSF25 GNG4 LIF DCBLD2 YRDC ARHGAP10 CCND1 SMOX SLC4A7 ERICH1 ABTB1 BCL9L PTGER4 RAB20 MT1G CHMP2B ADORA2B RRM2 MYADM DFFB ELFN2 RASSF1 ACTN1 STX11 CCDC107 PTS TCP10L CCM2 FAM135A MAP2K3 LYAR KRT18 NUMBL KCTD13 RGS17 DDX21 SLC9A3R1 ENC1 C9orf167 MGC102966 MOSPD1 COQ10B FRMD6 CAMSAP1 SDF2L1 ABHD6 C9orf89 OSGIN1 ELL UAP1 C3orf52 NKX3-1 MAD2L2 FAM83B FCHO1 FAM3C RNF24 NAMPT GKN2 LPCAT1 PRICKLE1 PLEKHM1 RRP9 GATAD2A GPR137B EDEM1 FHOD3 PHF10 RNF126 HBP1 LRRC8A MAP1LC3B SNX8 MCTP2 KIAA1539 TUBB2C UBR1 SATB2 FER PPP2CB ATXN1 C9orf169 TSSC4 TUBB4Q STX1A ARPC5L YAP1 C1orf116 GDPD3 IL18R1 FJX1 ANXA1 NT5DC3 CASP4 C5orf62 PANX1 TPBG TNFAIP3 PLEKHH3 UPP1 CYP24A1 CAP1 PPEF1 FAM126B C15orf39 C11orf24 MPZL2 STMN3 MPZL1 ACSL3 KPNA4 KRT8 ASB6 NT5E DPP9 ITPR3 SPHK1 CRY1 HIF1A IMP4 MFSD10 ZNF296 ATP2A2 SERTAD2 ITGA1 MBP RHEBL1 C1orf77 BTG3 S100A3 PPP1R16A IGHMBP2 FHL2 KIAA1804 PAQR5 CCNYL1 PTRF UBIAD1 EHD1 ISG20L2 SCNN1A TPRG1L CDC37 PRKCE HPCAL1 EXTL2 CITED1 LAMA4 RDH13 SLC1A5 PPAT MYO9B PNMA1 C10orf41 ADAMTSL5 ASGR1 PHLDB3 AKIRIN2 ETV4 SLC9A5 PPP1R13L ZNF335 C14orf129 GCAT SCNM1 SOCS3 KBTBD2 GJB3 C20orf20 FXR2 HRH1 NET1 HYAL3 MED4 DENND5A RRP15 RASGEF1A C16orf87 FZD10 TRMT1 TFE3 TOP1 CTRL CDC34 SLC2A13 LRRC3 HIC2 SLC6A6 SLK SMG5 TRIM16 BCL7B DOK7 GTF3C5 FSCN1 SHANK2 IPPK KRR1 EPHX4 ACOT9 ICAM3 PFDN2 BCL10 DPH2 TAF1B C19orf44 SAPS1 LOC652276 CYP27B1 MAPK13 BCL2L12 SRGN CD44 SERTAD3 BCAR1 FOXA1 ARID3A ZNF394 TRNP1 SPAG4 ST6GALNAC6 HIVEP1 BZW1L1 SELS NIP7 MORC3 DDX47 EIF5A2 KCNG3 PLEKHF1 DEDD2 ANXA5 LOC493754 FOSB PRRG1 KLF9 PID1 KRT15 MED26 FNDC3A PDE4D RABEPK TRMT61A RPS6KA3 DOCK5 CAPG IGSF8 URB2 MYD88 CD63 CCDC21 PRMT3 JOSD1 FGF11 HAUS2 CCDC68 RAB11FIP5 KHNYN CXorf40B PIM1 PINX1 TMEM87B FGD6 RRS1 DDX56 PIM3 ARHGDIA IRF9 HOXB8 PKM2 IRX5 PTGES WDR55 ATL1 GLDC EIF6 SIPA1L2 RBM9 MAPK6 SH3GL1 SIX1 RPF2 FTSJ3 RUSC2 MEX3D HOXB4 TPST2 ZNRD1 DSCR3 ZBED4 PRKAA2 POLR2F CLTB NXT1 ALKBH2 PPAN-P2RY11 TGDS NANS CCNE1 ZSWIM4 CSNK1D ERGIC1 EPHB4 ARNT2 CDC123 WDFY2 FBXO5 TP53BP2 EBNA1BP2 AATF |
| Illumina_SAM_DW_612  E4F1 ANAPC5 LOC400657 TMEM8B ZNF589 MIER1 KIAA1370 RMI1 C6orf211 ANGEL1 RARB SELO LMO1 RPS27L CC2D1A TSNAX KIAA0831 TYMS LSS RBBP9 COL1A1 MRPS27 CCDC28A NFYC CHM LIPT2 NAP1L1 TTC30A RABL5 GLRB SELT RNF135 TSPAN6 KCTD18 LZTFL1 FNBP1L KIAA1310 LOC440925 AK3 C6orf70 JAG2 CTPS2 WBP5 SPAG7 ST7 CTTNBP2 LYRM5 LRRC20 LPIN2 YIPF4 AEBP2 TRAK2 GPATCH1 CHDH KCTD21 FH RCCD1 ALG9 TRAM2 MLXIPL COL16A1 CENPK ADAT1 GAS8 TESK1 MMS19 PLCG1 KLHL8 SFRS7 ITGB3BP ALOX12B LOC81691 LACTB2 UBLCP1 TRIM68 GEMIN8 ZNF76 LACTB MXD3 TTF2 ZCRB1 FECH ZNF260 KLHL24 IPO9 HIST1H2AC PDIK1L SPINK5 DTL ANKRD26 VASH2 EPHX1 WIPF1 NCRNA00095 PGAP3 MYH10 C1QTNF6 ZC3H8 CMPK1 PPP1R3E PKD2 MCM6 BNIP3 MTA3 TMEM66 NR4A1 GPD1L ACAD11 CENPE HHLA3 DERA MORF4L1 STX16 BBS1 SRR MLF1IP MIOS PARP2 DNAJC19 FAM192A TMEM70 FAM111A ERMP1 ZNF285A DUS4L QPRT MRPS5 VRK1 MUDENG DDX28 EFNA4 SNORD25 LIN9 SMAD5 PIGS KIF21A ZC3H7A HNRPLL ARMCX2 CAMLG VPS54 SYNM OCEL1 OXA1L COASY GBF1 TMEM107 PTEN AFG3L2 GTF3C3 CEP70 CSRNP2 RFX5 POLD3 PMPCB JPH1 TOR2A DVL2 UBE4B FOXRED2 C2orf43 KLHL23 TRA2A NKD2 ATG10 C10orf104 CCDC111 DAZAP2 GTF3C2 WBP1 RANBP9 ZNF770 SUMF1 TIMM9 TMEM17 ZUFSP TCEAL8 ZNF621 HSDL1 ULK1 PRUNE C12orf47 MRPL35 IDH1 LRTOMT RAP2A KIAA1522 HIST1H4C RAB28 COQ7 MED25 C1orf115 PSMA2 C9orf123 CXorf57 RNF170 C19orf21 YTHDC2 ALDH3A2 SPAG16 TTC23 HDDC2 ALDH1L2 SMOC1 TRIM45 CDC7 HINT2 CLCNKA SMC2 C20orf177 RPAP3 TMED5 ADO HADH ZFAND5 PCMTD2 MRPS31 XRCC1 PRKCQ C2orf15 PDE8B NCOA4 NIPSNAP1 ZNF280D NASP APEH SALL2 C1orf59 C10orf57 ZNF512 THYN1 PLAGL2 HIST2H2BE PIGW JUND DEM1 PNPLA4 CTPS RUNDC3B UBXN4 AMZ2 ZNF664 NAT14 CCDC102A DBP RHOT1 RCN1 EPRS FAM173B CYP2U1 EFEMP1 UQCRC2 NR1H3 GOT1 IMPDH2 DNAJC15 SYNC C1RL SLC29A3 KIAA0319L NFIB CLTCL1 LOC401431 MLST8 NISCH ST3GAL3 C1orf66 TAF6L NKX3-2 N4BP2L2 RHOB FAM81A MMACHC DOLK PSMD4 BASP1 AP3M2 C22orf39 API5 PHF21A PIGC RPAP2 SEPP1 ISOC1 C21orf33 C18orf56 PRKAB2 SLTM PHOSPHO2 BAMBI PPRC1 ZNF33B ACACB SEMA6A CYR61 ZSCAN18 TP53 ZCCHC11 VPS36 C20orf30 IPP DMAP1 INTS3 SYNJ2BP MRPL15 ITFG3 VPS72 PLEKHA1 BET1 MCCC1 CDK5RAP3 RASL11A HNRPDL SAC3D1 CHAF1B GLOD4 TBX1 DYNC1LI2 FAM96A FAM8A1 ALG6 TSG101 ATRIP UFSP2 FAM45A KNTC1 DCPS PLSCR4 AMACR NFKBIA ZDHHC12 MAP2K5 TTC31 ABCA1 TAF9B SRP68 EFR3A INTS5 DENND5B KIAA1009 DDX59 POLR3C HDHD2 RFX1 SLC25A23 RTN4IP1 KPNA6 RBM45 SPG11 CYB5D2 RPUSD3 KIF15 MTR CDC23 MSRB2 STAT2 NSUN4 CBR4 HAUS4 SRBD1 ASTE1 RAB32 FAM35A CTDSPL ZSCAN21 PLK1S1 ALDH9A1 C22orf13 MRPS11 IL10RB RBM17 THNSL1 CDCA7L SCOC WDR75 MMP24 ADA NICN1 FAM149A C5orf54 FAM175A FBXL16 PEX11G ZFX PPAPDC3 VPS39 DEPDC1 WDR70 WNK1 PIGY BIVM SMARCAL1 KIAA0776 CCDC53 NEK2 MXD4 C18orf55 MRPS23 AKR1C3 WDR19 SF4 TP53INP1 TSC22D3 PCM1 LSM14A C1orf25 C20orf108 PLD1 CCDC25 C21orf125 ZNF362 ALDH3B1 C14orf93 ZBED5 SETMAR TBC1D9 ENO3 OAZ2 MPPE1 SPATA7 C4orf34 NUSAP1 FASTK ATP6V1E2 ARMC1 COPS4 ZFP90 ARL2BP COQ2 COX15 CCDC113 BBS2 ASH2L C7orf25 HOXB13 HMMR VPS33B C1orf123 SH3BGRL2 PAN2 PRKAA1 NLRX1 KDM3B CPSF3 KIAA0528 ASB13 CRB1 SCCPDH CD99L2 SLC25A12 PAAF1 FANCL MUTYH SACM1L CHURC1 RNF141 PHTF1 PPL DDX23 UNKL DIO2 KLF2 C7orf58 PRKD3 TWIST1 KLHL9 WDR44 CCNG1 AASDHPPT KANK2 RBM5 RHOU H1FX DSN1 TMEM101 MSTP2 TP53INP2 PPCS BRCA1 RAB22A NT5DC1 CEBPD TMEM42 EPM2AIP1 C16orf75 CCDC85B REEP5 DLGAP5 ACOT1 SLC35A5 IVNS1ABP SGPP1 PAFAH1B1 NGFRAP1 RDH14 DHRS3 ZNF337 FAM102B BRD3 GALM ATG4C FOXO4 RDH11 KLHL3 PHIP SCARA3 HDAC4 GPR37 HSPA2 COQ10A TUBGCP5 CTGF OMA1 SNCA HIST2H4B TLCD1 ASXL1 PSMD10 TMEM138 CPT2 SKP2 CCNA1 NEK8 WNT5A CCDC14 ARHGEF17 PHLDB1 EIF4A2 SNX19 PRMT6 DPH5 PRMT7 HS1BP3 ACP1 KDELC2 POLR3GL DNAL4 BRMS1L PIR PEX11B RNF20 ABCB7 SNX2 PRSS23 C5orf33 CENPF ABHD4 CABC1 PPAPDC2 EEF2K TJP3 KIF11 UBAP2L FLYWCH2 C14orf147 STRADB COG2 ID3 CITED4 WDR60 PIGV MED20 GEMIN6 PSMD7 ARV1 DCAF8 ASPM FOXQ1 NUDT18 VIPR2 HMGCL PRIM1 DIS3L ZNF467 KLHDC2 ARID5B CRBN FAM175B NR4A2 COQ5 TGFBR3 COQ9 ELF3 FSTL3 XPC DDIT4L MOSC1 RAB26 MN1 C5orf13 DUSP3 RHOV ECHDC3 SLC11A2 KCNIP3 SALL4 CITED2 CCDC117 GAS1 ROR2 ZNF323 CYBASC3 C22orf29 COBLL1 PDXK TNS3 RDH10 TMEM187 LMO4 ZXDB FOS TNFSF10 |
