# Supplementary material for: Multiple platform assessment of the EGF dependent transcriptome by microarray and deep tag sequencing analysis
Source: BMC Genomics. 2011 Jun 23;12:326. doi: 10.1186/1471-2164-12-326 (PMC3141672; doi:10.1186/1471-2164-12-326)
Supplement: Additional file 3 — Table S2. Table of cross platform GSEA enrichment scores and significance values (provided as word file). [file 1471-2164-12-326-S3.DOC]

**Platform Test Set Comparison SIZE ES NES NOM p-val FDR q-val**

**________________________________________________________________________________**

**Agilent** Upregulated

AGILENT 6h EGF vs Control 1084 0.990 4.150 0.000 0.000

OPERON 6h EGF vs Control 412 0.910 3.590 0.000 0.000

ILLUMINA 6h EGF vs Control 489 0.890 3.580 0.000 0.000

Downregulated

AGILENT 6h EGF vs Control 1187 -0.990 -4.210 0.000 0.000

OPERON 6h EGF vs Control 325 -0.860 -3.300 0.000 0.000

ILLUMINA 6h EGF vs Control 573 -0.830 -3.400 0.000 0.000

**________________________________________________________________________________**

**Operon** Upregulated

AGILENT 6h EGF vs Control 1063 0.810 3.920 0.000 0.000

OPERON 6h EGF vs Control 415 0.980 4.420 0.000 0.000

ILLUMINA 6h EGF vs Control 487 0.840 3.830 0.000 0.000

Downregulated

AGILENT 6h EGF vs Control 1153 -0.730 -3.790 0.000 0.000

OPERON 6h EGF vs Control 335 -0.980 -4.660 0.000 0.000

ILLUMINA 6h EGF vs Control 571 -0.730 -3.640 0.000 0.000

**________________________________________________________________________________**

**Illumina** Upregulated

  AGILENT 6h EGF vs Control 1034 0.900 3.400 0.000 0.000

  OPERON 6h EGF vs Control 406 0.910 3.220 0.000 0.000

  ILLUMINA 6h EGF vs Control 493 0.970 3.490 0.000 0.000

  Downregulated

  AGILENT 6h EGF vs Control 1138 -0.840 -3.180 0.000 0.000

  OPERON 6h EGF vs Control 323 -0.850 -2.950 0.000 0.000

  ILLUMINA 6h EGF vs Control 578 -0.970 -3.540 0.000 0.000

**________________________________________________________________________________**
