# Supplementary figures and images for: Multiple platform assessment of the EGF dependent transcriptome by microarray and deep tag sequencing analysis
Source: BMC Genomics. 2011 Jun 23;12:326. doi: 10.1186/1471-2164-12-326 (PMC3141672; doi:10.1186/1471-2164-12-326)

## Slide 1
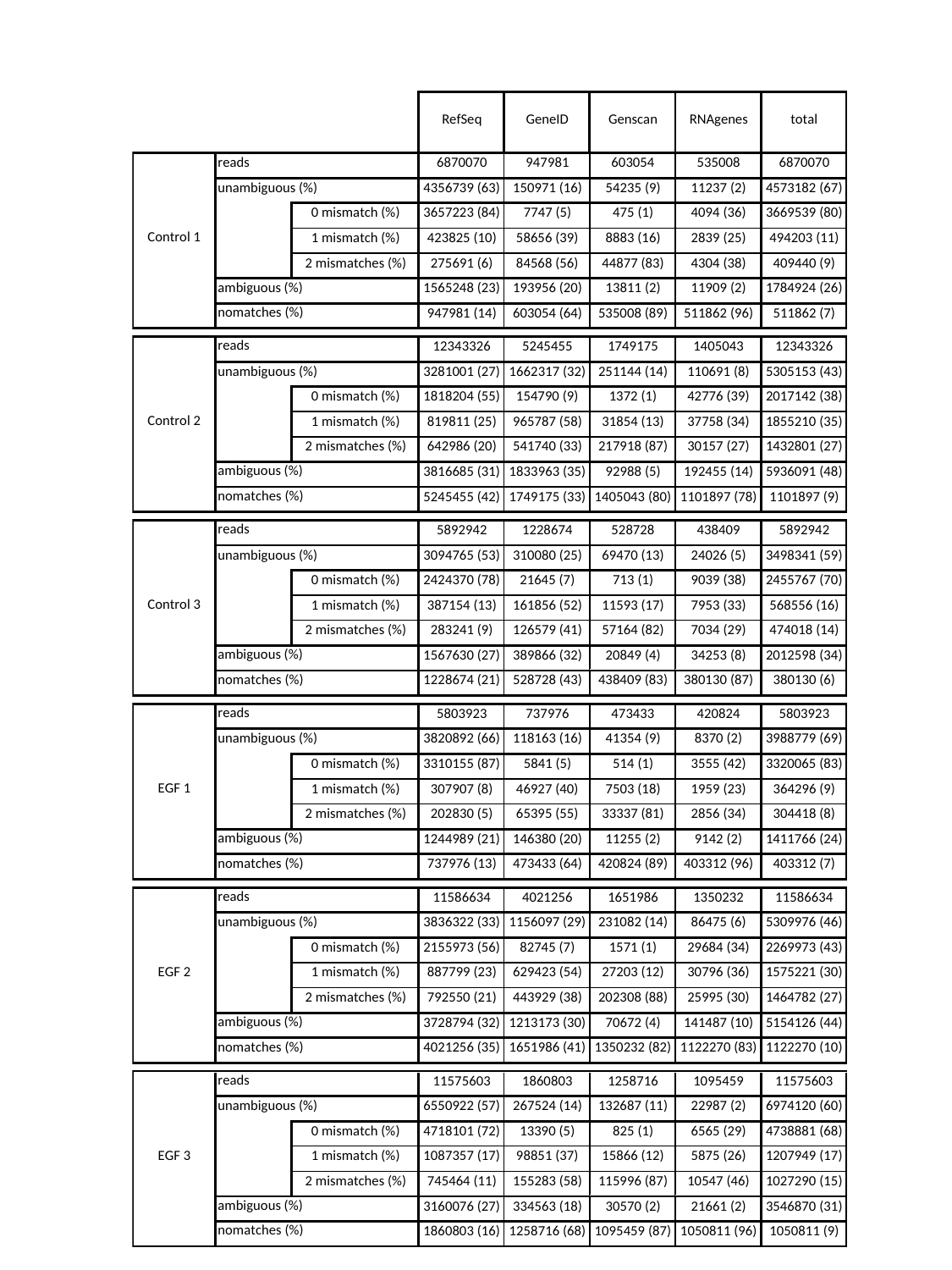

#

Supplement: Additional file 5 — Table S4. Table of reads generated by the DGE pipeline for each of the runs. Summary table of read mapping statistics generated by the DGE pipeline for each of the runs. [file 1471-2164-12-326-S5.PPT]

## Slide 1
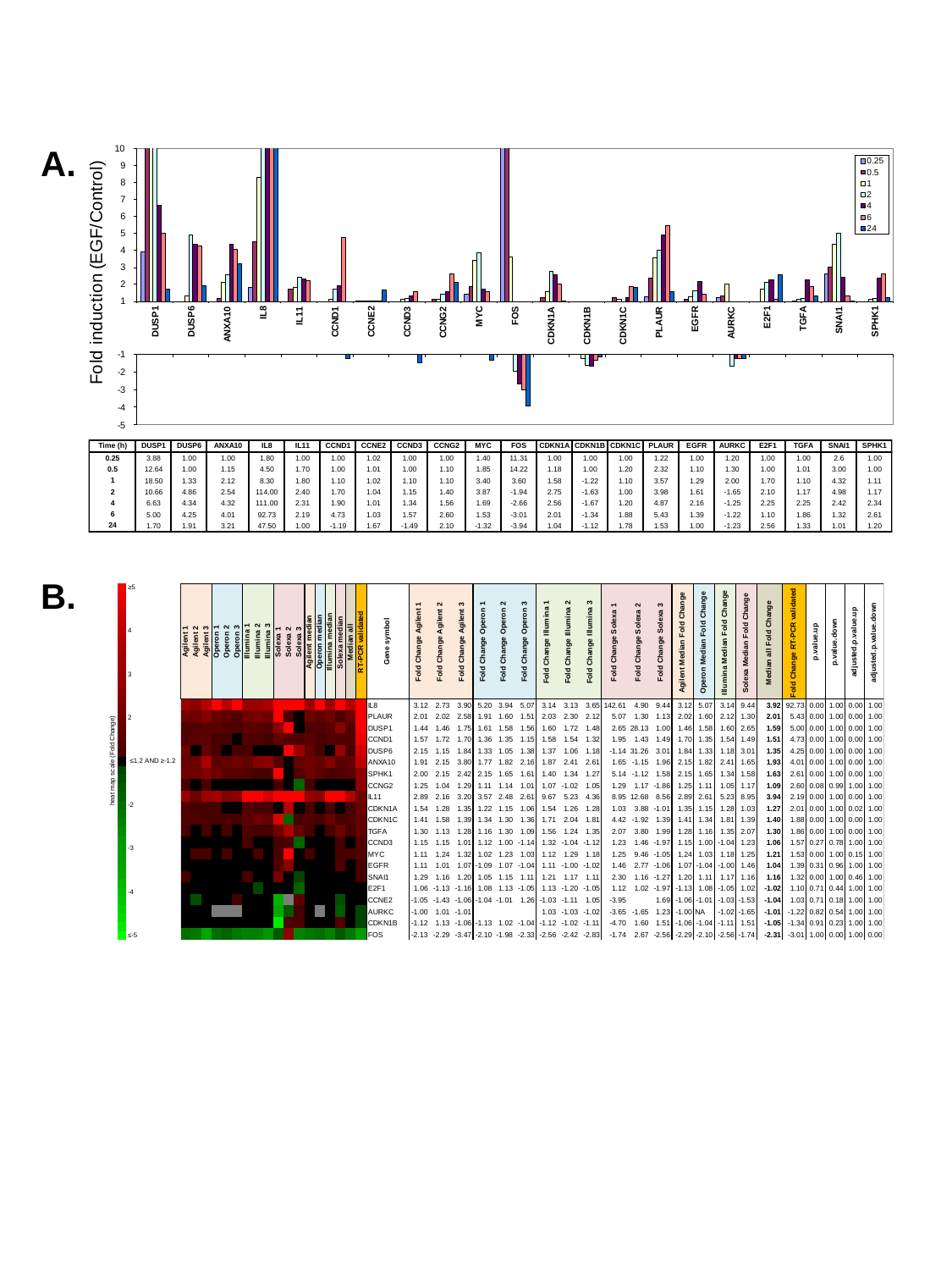

A.
B.

Supplement: Additional file 8 — Figure S2. Time course RT-qPCR analysis of potential EGF-regulated mRNAs. Total RNA samples from serum-starved HeLa cells stimulated with EGF at the indicated times (15 min to 24 h) were subjected to quantitative real-time PCR (see Methods for details). Data represent mean fold induction of at least two independent experiments. SFA3 was used as the reference. (A) The upper panel shows the graphical representation. (B) RT-qPCR Fold Changes and corresponding Fold Changes derived from the three microarray platforms and by ultrasequencing. [file 1471-2164-12-326-S8.PPT]

## Slide 1
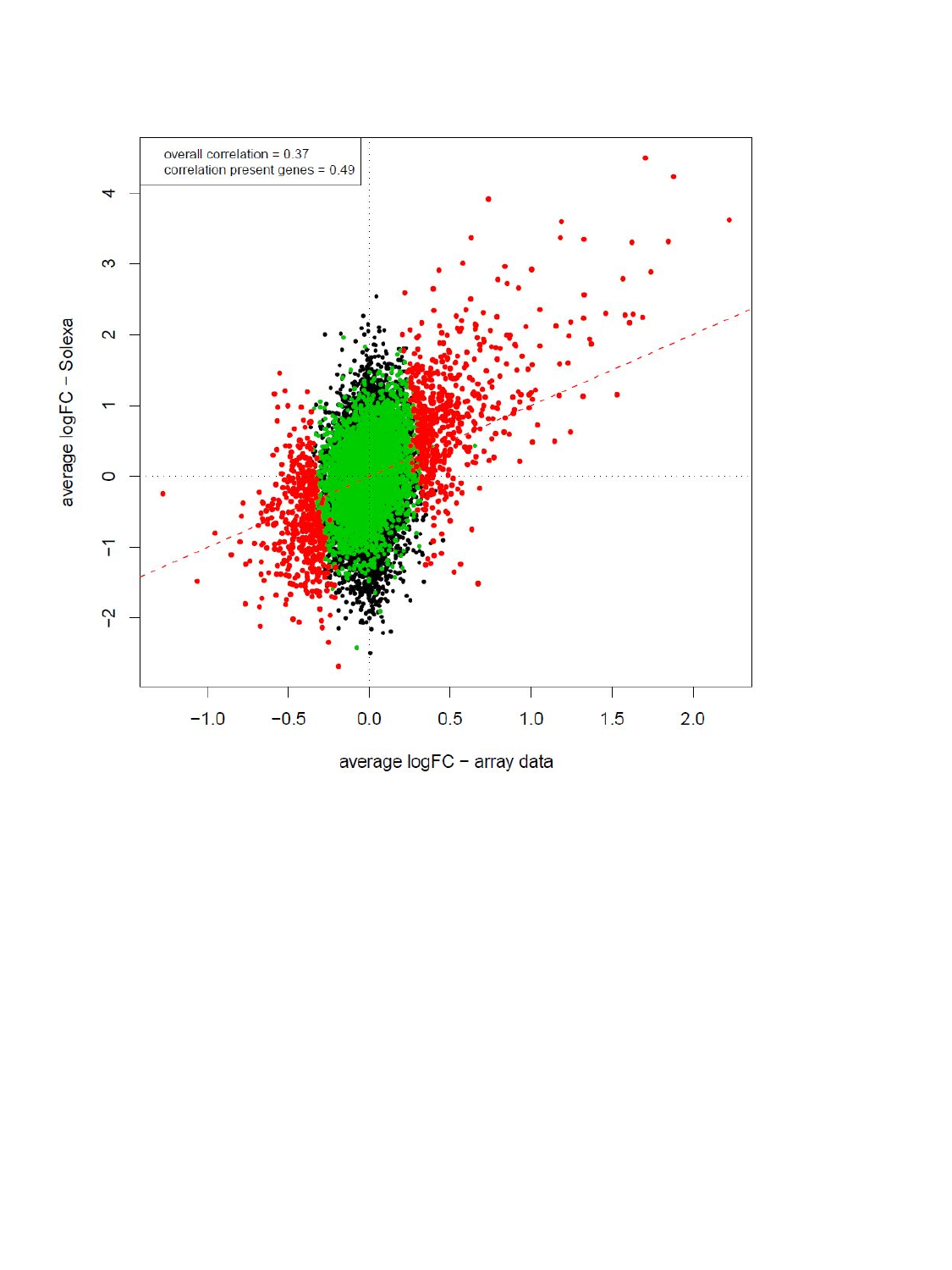

Supplement: Additional file 9 — Figure S3. Correlation plot between DGE and microarray log2ratio values. Comparison of estimated log2ratios from DGE (Y-axis) and the average of all three microarray platforms (X-axis). We consider only genes that were interrogated using all platforms and genes with a mean number of counts across lanes greater than 0. Genes with counts greater than 32 reads in all samples (colored red or green) or less than 32 reads (black) in at least one sample are shown. (Red dots) Genes called differentially expressed based on DGE data at a 10% FDR by RankProd. (Green dots) Genes not called differentially expressed but above 32 counts. (Inset box) Correlation between technologies is higher when considering only genes above the 32 count detection level than when all genes are included. [file 1471-2164-12-326-S9.PPT]

## Slide 1
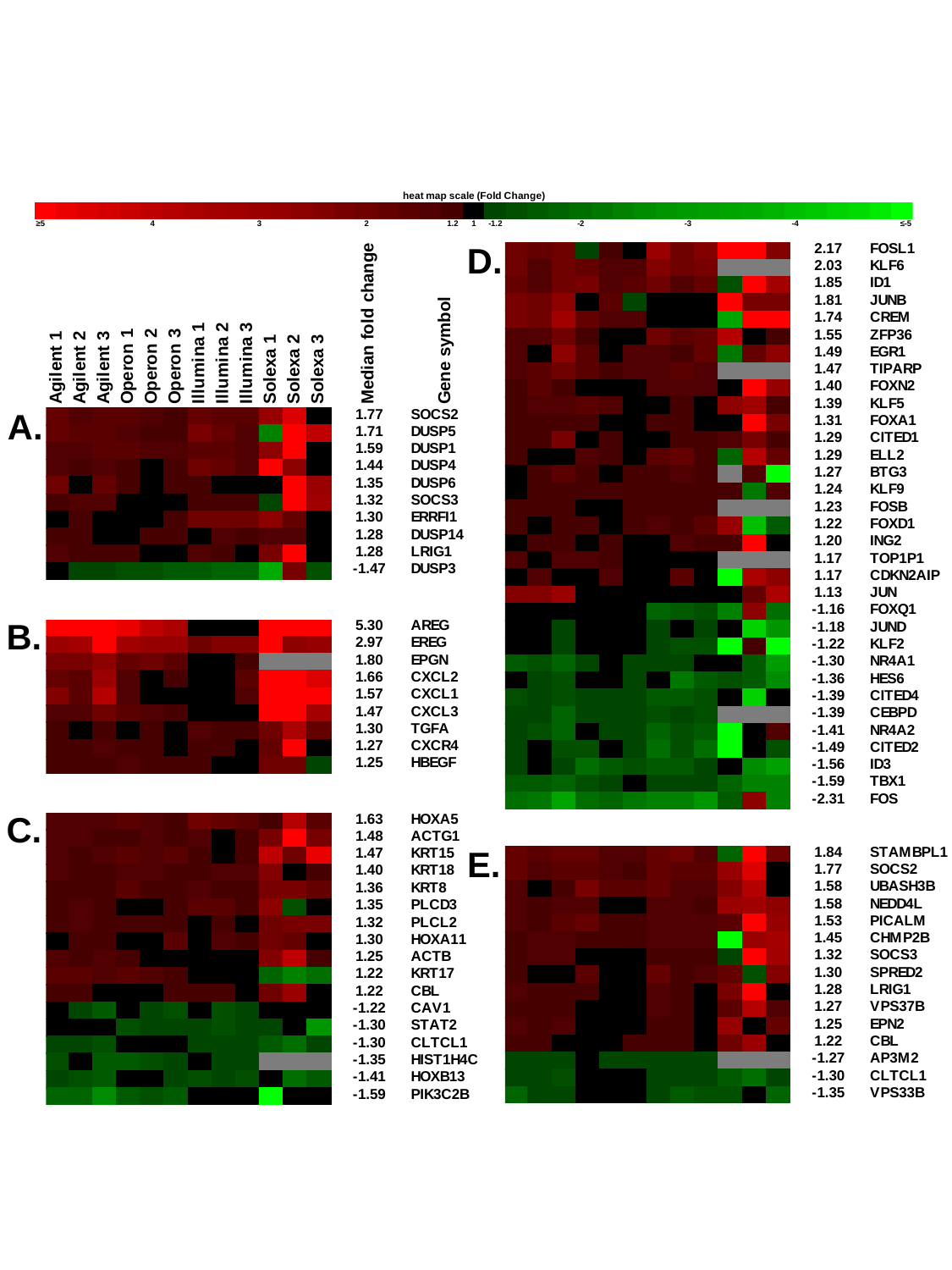

D.
A.
B.
C.
E.

Supplement: Additional file 10 — Figure S4. Heat maps of genes found regulated at 6 h after EGF treatment of HeLa cells in our study and known to be related to EGF signaling. Some genes detected in a subset of all platforms are also included for the sake of completion. (A) Modulators of EGF signaling; (B) non-EGF agonists of EGFR and cytokines linked to the EGF family locus on chromosome 4q13.3; (C) EGF-interacting and related proteins; (D)genes described as early and delayed early response to EGF including DNA and RNA binding proteins; and (E) components of the ERBB receptor endocytosis and intracellular trafficking complexes. [file 1471-2164-12-326-S10.PPT]

## Slide 1
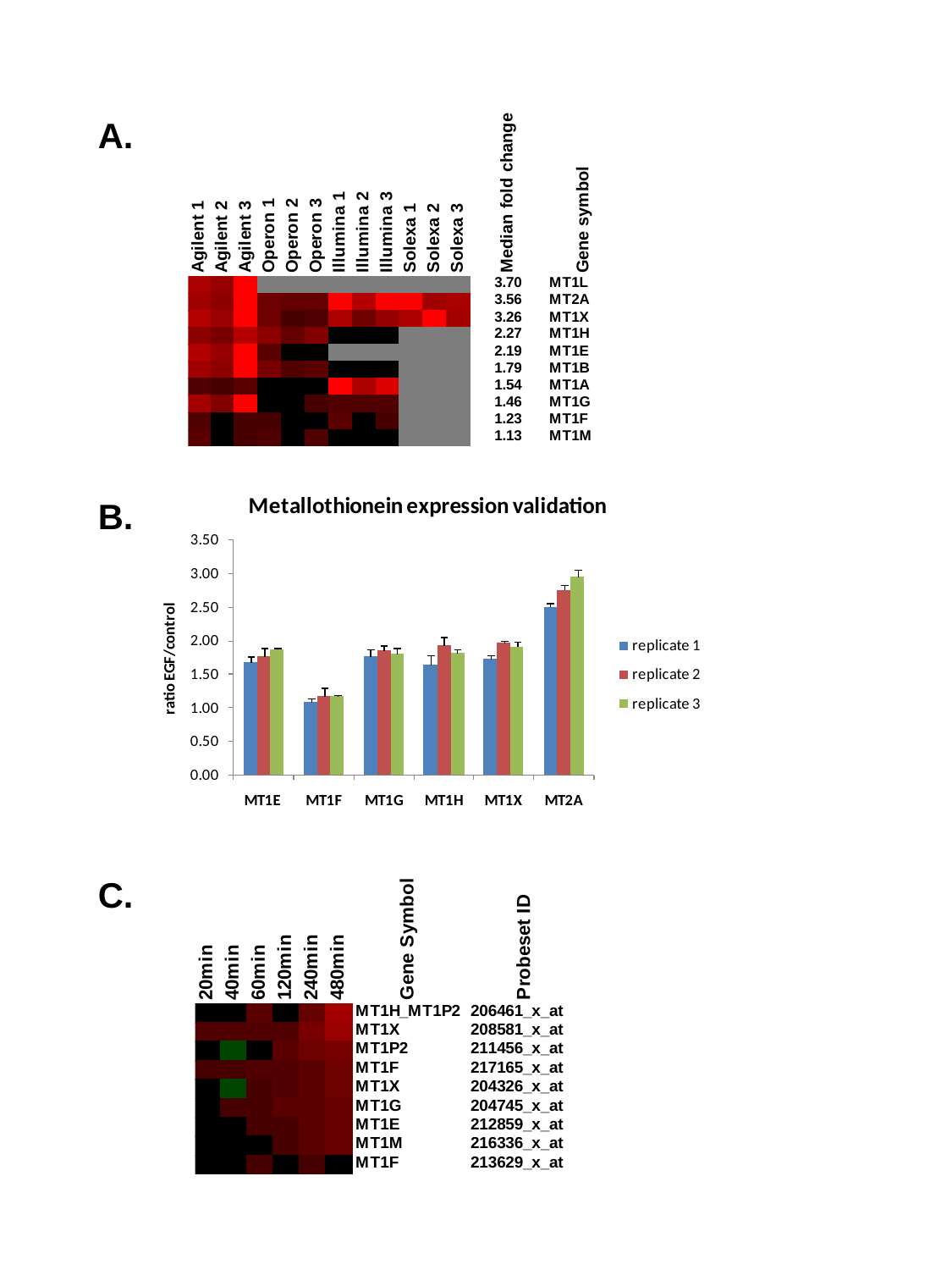

A.
B.
C.

Supplement: Additional file 11 — Figure S5. Metallothionein gene expression after EGF treatment. Log2ratio of EGF-treated versus untreated heat maps of metallothionein gene expression after EGF treatment in (A) HeLa cells at 6 h as determined in this study using Agilent, Operon, and Illumina microarrays, and DGE sequencing; (B) RT-qPCR for 6 metallothionein family members, (C) metallothioneins in HeLa cells in the time course study by Amit et al using the Affymetrix platform, without replication (relative log2ratios obtained by log2intensity subtraction of the 0 time point value from each time point). [file 1471-2164-12-326-S11.PPT]

## Slide 1
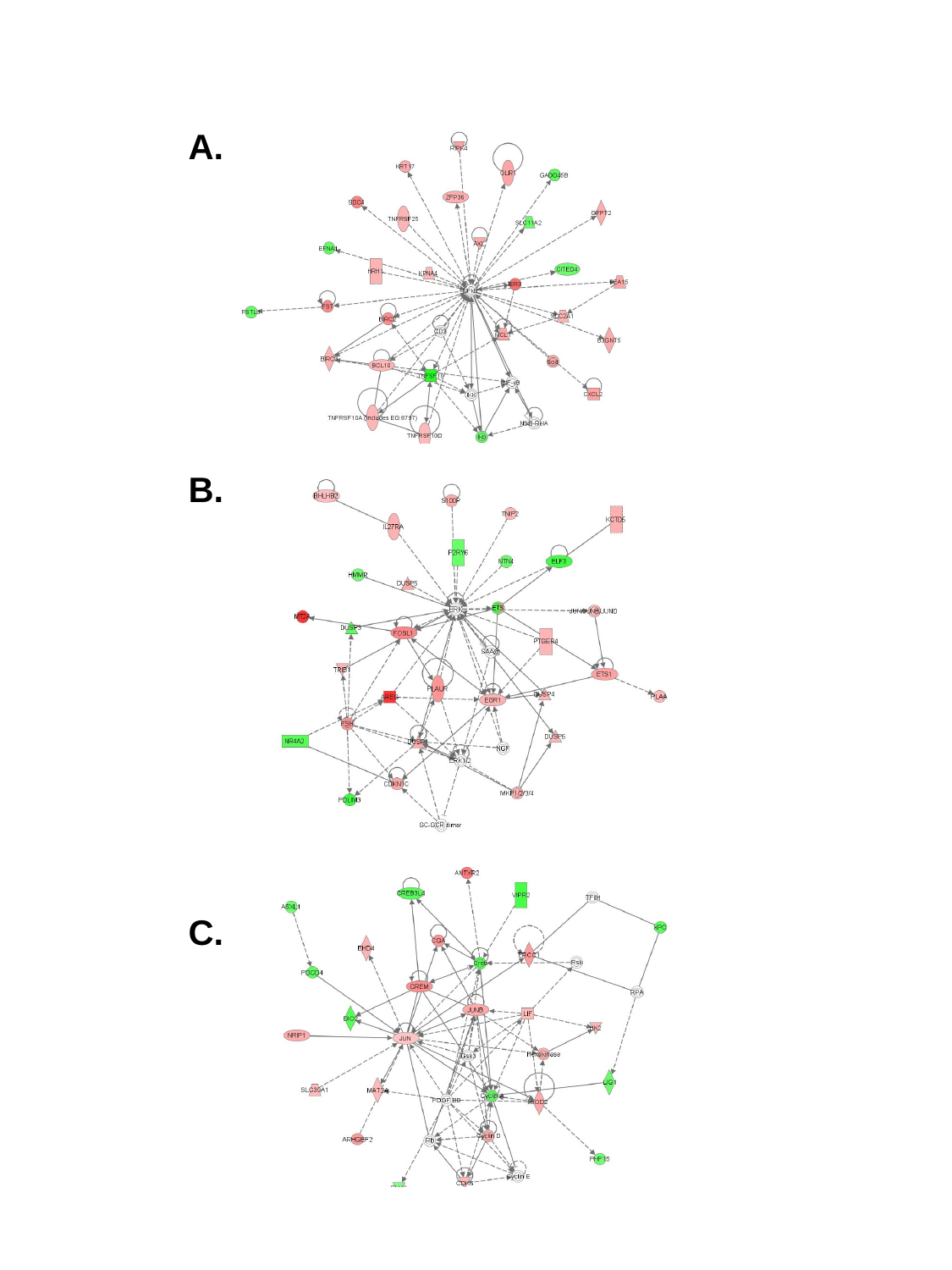

A.
B.
C.

Supplement: Additional file 12 — Figure S6. Pathway analysis based on the Ingenuity Pathway Knowledge base. The three best ranked networks derived from EGF-regulated genes as determined by the RankProd test were (A) Cell Death, Embryonic Development, Renal and Urological Disease (B) Amino Acid Metabolism, Post-Translational Modification, Small Molecule Biochemistry and (C) Cell Cycle, Cancer, Cardiovascular System Development and Function. Upregulated genes are indicated by red symbols and down-regulated genes by green symbols. The shape of the node denotes the main function of the protein encoded by the gene. Smooth lines indicate interaction between the products of the genes; dashed lines indicate an indirect interaction and lines with an arrow indicate an "acts on" relationship. Regulated genes are shown as grey boxes; non-regulated genes associated with the regulation of some of these genes are shown as white. [file 1471-2164-12-326-S12.PPT]

## Slide 1
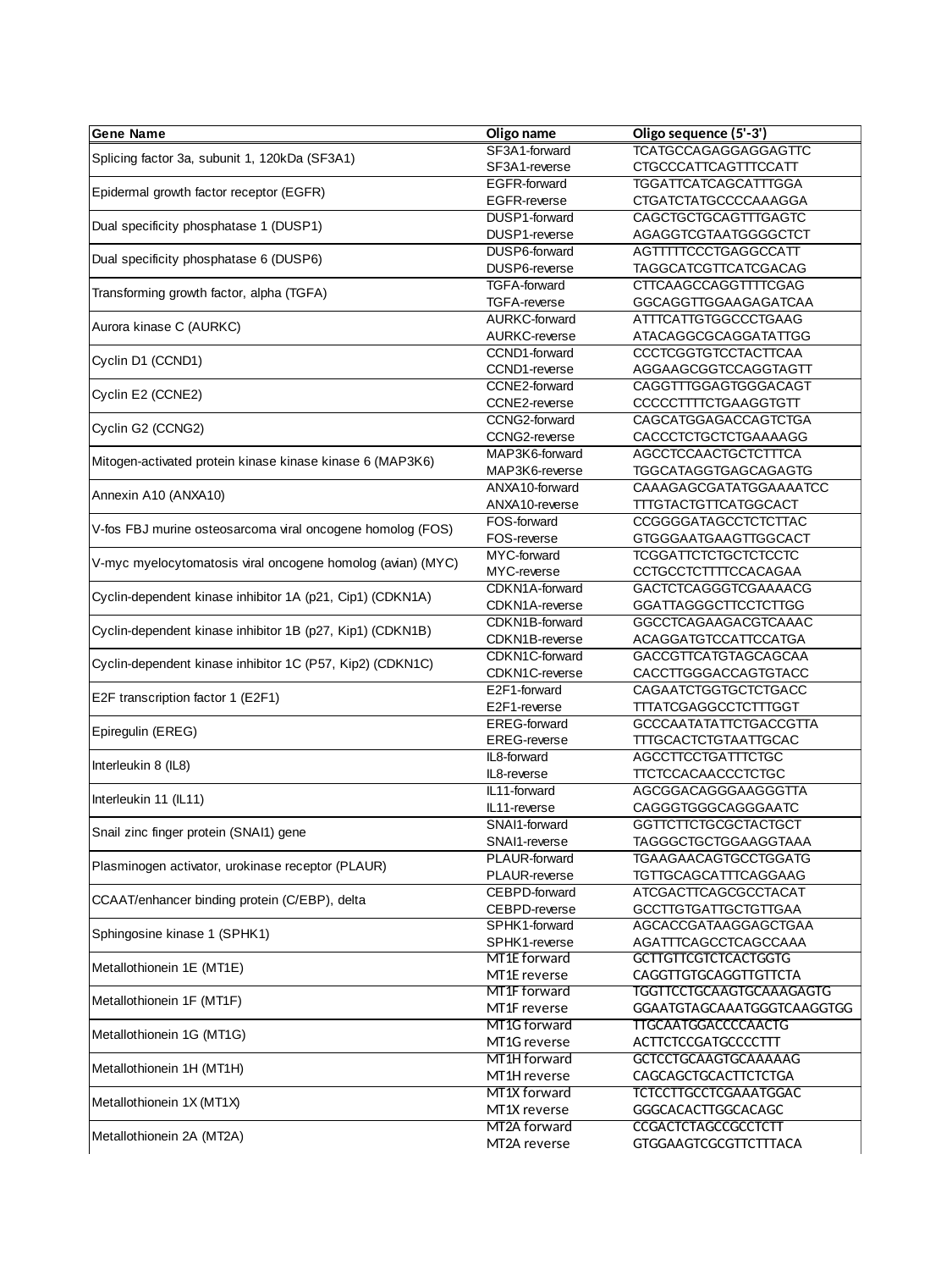

Supplement: Additional file 14 — Table S8. List of primers used in this study. [file 1471-2164-12-326-S14.PPT]
